# Association of Neighborhood Racial and Ethnic Composition and Historical Redlining With Built Environment Indicators Derived From Street View Images in the US

**DOI:** 10.1001/jamanetworkopen.2022.51201

**Published:** 2023-01-18

**Authors:** Yukun Yang, Ahyoung Cho, Quynh Nguyen, Elaine O. Nsoesie

**Affiliations:** 1 Center for Antiracist Research, Boston University, Boston, Massachusetts; 2 Department of Political Science, Boston University, Boston, Massachusetts; 3 Department of Epidemiology and Biostatistics, School of Public Health, University of Maryland, College Park; 4 Department of Global Health, School of Public Health, Boston University, Boston, Massachusetts

## Abstract

**Importance:**

Racist policies (such as redlining) create inequities in the built environment, producing racially and ethnically segregated communities, poor housing conditions, unwalkable neighborhoods, and general disadvantage. Studies on built environment disparities are usually limited to measures and data that are available from existing sources or can be manually collected.

**Objective:**

To use built environment indicators generated from online street-level images to investigate the association among neighborhood racial and ethnic composition, the built environment, and health outcomes across urban areas in the US.

**Design, Setting, and Participants:**

This cross-sectional study was conducted using built environment indicators derived from 164 million Google Street View images collected from November 1 to 30, 2019. Race, ethnicity, and socioeconomic data were obtained from the 2019 American Community Survey (ACS) 5-year estimates; health outcomes were obtained from the Centers for Disease Control and Prevention 2020 Population Level Analysis and Community Estimates (PLACES) data set. Multilevel modeling and mediation analysis were applied. A total of 59 231 urban census tracts in the US were included. The online images and the ACS data included all census tracts. The PLACES data comprised survey respondents 18 years or older. Data were analyzed from May 23 to November 16, 2022.

**Main Outcomes and Measures:**

Model-estimated association between image-derived built environment indicators and census tract (neighborhood) racial and ethnic composition, and the association of the built environment with neighborhood racial composition and health.

**Results:**

The racial and ethnic composition in the 59 231 urban census tracts was 1 160 595 (0.4%) American Indian and Alaska Native, 53 321 345 (19.5%) Hispanic, 462 259 (0.2%) Native Hawaiian and other Pacific Islander, 17 166 370 (6.3%) non-Hispanic Asian, 35 985 480 (13.2%) non-Hispanic Black, and 158 043 260 (57.7%) non-Hispanic White residents. Compared with other neighborhoods, predominantly White neighborhoods had fewer dilapidated buildings and more green space indicators, usually associated with good health, and fewer crosswalks (eg, neighborhoods with predominantly minoritized racial or ethnic groups other than Black residents had 6% more dilapidated buildings than neighborhoods with predominantly White residents). Moreover, the built environment indicators partially mediated the association between neighborhood racial and ethnic composition and health outcomes, including diabetes, asthma, and sleeping problems. The most significant mediator was non–single family homes (a measure associated with homeownership), which mediated the association between neighborhoods with predominantly minority racial or ethnic groups other than Black residents and sleeping problems by 12.8% and the association between unclassified neighborhoods and asthma by 24.2%.

**Conclusions and Relevance:**

The findings in this cross-sectional study suggest that large geographically representative data sets, if used appropriately, may provide novel insights on racial and ethnic health inequities. Quantifying the impact of structural racism on social determinants of health is one step toward developing policies and interventions to create equitable built environment resources.

## Key Points

**Question:**

What is the association of built environment indicators derived from online street-level images with racial and ethnic composition of neighborhoods?

**Findings:**

In this cross-sectional study, predominantly White neighborhoods overall had fewer dilapidated buildings, fewer non–single family homes, fewer single-lane roads, and more green space compared with neighborhoods with residents of multiple races and ethnicities, predominantly Black residents, and predominantly minoritized racial or ethnic group residents other than Black.

**Meaning:**

These findings suggest that improved large-scale data on built environment features may provide better documentation of neighborhood inequalities and improve understanding of how structural racism manifested through the built environment is associated with poor health outcomes.

## Teaser

This cross-sectional study uses built environment indicators derived from street-level images to quantify racial and ethnic disparities in built environment resources and assess how the built environment mediates the association between neighborhood racial and ethnic composition and poor health outcomes.

## Introduction

Neighborhoods and the built environment are important social determinants of health. The built environment, defined as “human-made space in which people live, work and recreate,” has been shaped by political and cultural factors in the US.^1,2,3^ For example, during the 1930s, the Home Owners’ Loan Corporation (HOLC) created color-coded maps to delineate areas deemed risky for investments based on overtly racist criteria. Areas were graded as follows: A represented best (green); B, still desirable (blue); C, definitely declining (yellow); and D, hazardous (red).^4,5,6^ The HOLC maps encouraged disinvestment in neighborhoods in which residents were predominantly members of racial and ethnic minority groups (areas C or D) while directing investment to wealthy and predominantly White neighborhoods (areas A or B).^7,8,9,10^ The racial, ethnic, and economic segregation, disinvestment, and discrimination created by redlining policies remain in several cities today.^4,5,6,7^ These policies have been associated with present-day health disparities, including preterm births, asthma, and mortality.^11,12,13,14^ Research has established that unequal distribution and access to resources that promote health and well-being have created significant differences in health outcomes.^15,16,17^

Studies have also shown associations between the presence or absence of specific built environment elements and certain health outcomes. Associations between neighborhood racial and ethnic composition and health are not due to biological differences between racial and ethnic groups. Rather, these disparities are due to policies and systems that uphold structural racism, leading to differential access to resources that promote health and well-being. Green spaces or greenery in neighborhoods, for example, have been extensively studied and associated with better mental, sleep, and cardiovascular health^18,19,20,21,22^ and a lower likelihood of coronary artery disease, hypertension, and diabetes.^23,24^ Other research has postulated that the presence of green space encourages recreational walking and social coherence and thus contributes to better overall health outcomes.^19^ Conversely, abandoned buildings and vacant land have been associated with poor mental and physical health.^25^ Individuals living in neighborhoods with more dilapidated buildings have higher rates of hospitalization for asthma in New York City.^26^ However, research is sparse on built environment elements (such as dilapidated buildings, crosswalks, single-lane roads) that are challenging to measure.

Neighborhood studies often collect data from manual audits, which tend to be limited in scale, generalizability, and a broader understanding of the association among the built environment, health, and neighborhood racial and ethnic composition. In-person or manual audits of neighborhood characteristics are resource costly and time consuming and can be inconsistent between studies depending on the measures and instruments used. In contrast, neighborhood indicators extracted from satellite and online street-level images have been shown to be useful for studying health and socioeconomic outcomes.^23,27,28^ Previous studies^23,27,29^ have used street-level image data to examine the association between built environment characteristics and health but have not focused on racial and ethnic inequities. In this cross-sectional study, we used built environment indicators derived from millions of street-level images to (1) quantify racial and ethnic disparities in built environment resources and (2) quantify how the built environment mediates the association between neighborhood racial composition and health outcomes.

## Methods

### Data and Measurements

In this cross-sectional study, we focused our analysis on urban regions and defined neighborhoods as census tracts. Urban classification of census tracts was based on the Rural-Urban Commuting Area Codes data set from the Economic Research Service, US Department of Agriculture.^30^ We focused on the census tracts classified as metropolitan areas (codes 1-3). The study followed the Strengthening the Reporting of Observational Studies in Epidemiology (STROBE) reporting guideline except in the selection of participants. The data and records used in the study were collected by third parties. The study was deemed exempt from review and the requirement for informed consent by the Boston University Institutional Review Board because all the data used were publicly available.

### Built Environment Characteristics

We used data described by Nguyen and colleagues.^23,27,29^ The data consisted of 164 million images extracted from Google Street View’s Application Programming Interface from November 1 to 30, 2019. Convolutional neural networks—the state-of-art model for computer vision tasks—were used to identify objects in the collected images. All models were trained and hypertuned by splitting the data into a training set and validation set using an 80:20 ratio for best model performance. eAppendix 3 in Supplement 1 provides additional information on image processing.

To produce neighborhood-level indicators, the binary indicators of each image were aggregated at the census tract level. The aggregated data indicated the percentage of all images in the tract that contain these built environment elements. The resulting data set consisted of 11 built environment indicators: dilapidated buildings, 2 or more cars, chain link fences, street signs, streetlights, green spaces, crosswalks, non–single family homes, single-lane roads, visible wire, and sidewalks. The data set covered 72 311 census tracts across the US.

We selected 5 indicators after conducting an exploratory data analysis to ensure the built environment indicators used were not overlapping, that there was research linking the indicator to health, that the data were not sparse, and that the indicator was easy to interpret. We focused on dilapidated buildings, green spaces, crosswalks, non–single family homes, and single-lane roads.

### Neighborhood Characteristics

We obtained data for 6 single racial and ethnic categories, namely American Indian or Alaska Native, Hispanic (of any race), Native Hawaiian or other Pacific Islander, non-Hispanic Asian (hereinafter referred to as Asian), non-Hispanic Black (hereinafter referred to as Black), and non-Hispanic White (hereinafter referred to as White) from the 2019 American Community Survey (ACS) 5-year estimates.^31^ We assessed neighborhood disparities in built environment indicators using 2 approaches: racial and ethnic majority tracts and neighborhood topology and HOLC grades. We categorized the racial and ethnic majority in a neighborhood using the same approach as Gibbons^32^:

- Predominantly Black: Census tracts with a population of at least 50% Black residents and less than 20% of the second largest racial or ethnic group.
- Predominantly White: Census tracts with a population of at least 60% White residents and less than 20% of any racial or ethnic minority group.
- Predominantly minoritized racial or ethnic group other than Black: Census tracts with a population of at least 50% of any members of racial and ethnic minority groups other than Black (including American Indian or Alaska Native, Asian, Hispanic, and Native Hawaiian or other Pacific Islander), and less than 20% of the second largest racial or ethnic group.
- Unclassified: Census tracts did not correspond with any of the classes above.

For the analysis using HOLC grades, we used the redlining census tract crosswalk data from the Mapping Inequality files.^33^ There are challenges to matching current census tracts to HOLC grades; about 60% of 2010 census tracts cross HOLC areas with multiple grades.^34^ We adopted the approach used by Krieger et al.^12^ For each census tract, we determined the percentage of the geographical area assigned to each grade. Then, we assigned the grade representing at least 50% of the area to the tract. If no HOLC grade was assigned to at least 50% of the census tract, the census tract was labeled “other.” This resulted in 2381 D, 3847 C, 1562 B, 425 A, and 6796 other grades. The remaining 44 228 census tracts could not be assigned a grade. eAppendix 7 in Supplement 1 provides additional information.

### Socioeconomic Indicators

We obtained covariates pertinent to the built environment indicators and health outcomes from the 2019 ACS.^31^ These included median household income, percentage of residents with a bachelor’s degree, median age, percentage of residents who were female, percentage of residents with health insurance (hereafter referred to as percent insured), percentage of owner-occupied housing, percentage of vacant housing, and percentage of residents who were a single female head of household with children.

### Health Outcomes

For health outcomes, we used the 2020 Population Level Analysis and Community Estimates (PLACES) data from the Centers for Disease Control and Prevention.^35^ PLACES provides census tract estimates for chronic disease risk factors, health outcomes, and clinical preventive services for all 50 states and Washington, DC, that have a Census 2010 population of 50 or more. The data are from the 2018 Behavioral Risk Factor Surveillance System that includes all survey respondents 18 years and older. Based on known associations between the built environment and health outcomes, we selected the following outcome variables: sleeping problems (defined as a model-based estimate of crude prevalence of sleeping <7 h per night), diabetes prevalence (model-based estimate of crude prevalence of diagnosed diabetes outside of pregnancy), and asthma prevalence (model-based estimate of crude prevalence of current asthma).^22,24,26,36,37^

### Statistical Analysis

Data were analyzed from May 23 to November 16, 2022. All statistical analyses were conducted using R, version 4.2.0 (R Project for Statistical Computing) using the lme4,^38^^(p4)^ cluster,^39^ and mediation^40^ packages, and statistical significance was assessed at 2-sided α = .05. We conducted exploratory analysis to assess the associations between the various variables in our datasets (eAppendix 1 in Supplement 1). We performed multilevel linear regression analyses using the built environment indicators as dependent variables and neighborhood racial topology as the independent variable. We used the predominantly White neighborhood as the reference group. The reason for using multilevel regression was to account for the clustering that might happen at the census tract level; the baseline level of the value for our variables might differ from state to state. We used the socioeconomic indicators as covariates. We also replicated the same analysis using the HOLC grades with neighborhoods assigned grade A as the reference group. A sensitivity analysis showed that our results were robust across varying definitions of neighborhood racial topology (eAppendix 8 in Supplement 1).

To study how structural racism within the built environment indicators mediates the association between neighborhood racial composition and adverse health outcomes, we constructed a series of multilevel mediation models (eAppendix 2 in Supplement 1). The mediation was conducted using a model-based approach.^41^ First, we fit a mediator model where the potential mediator—built environment—was estimated by our treatment variable, the neighborhood racial composition. Then, we constructed the model for our outcome variable—separately for each of the health outcome variables—where the independent variables are the neighborhood racial composition and the built environment. The 3 types of neighborhood topology comparisons were (1) predominantly Black neighborhoods referencing predominantly White neighborhoods, (2) predominantly minoritized racial or ethnic neighborhoods other than Black neighborhoods referencing predominantly White neighborhoods, and (3) unclassified neighborhoods referencing predominantly White neighborhoods. Each model included the same covariates and a random intercept for state, consistent with our multilevel linear regression models.

## Results

The data set included 59 231 urban census tracts representing all US states and Washington, DC (Figure). The demographic characteristics across census tracts are provided in Table 1.

**Figure.**
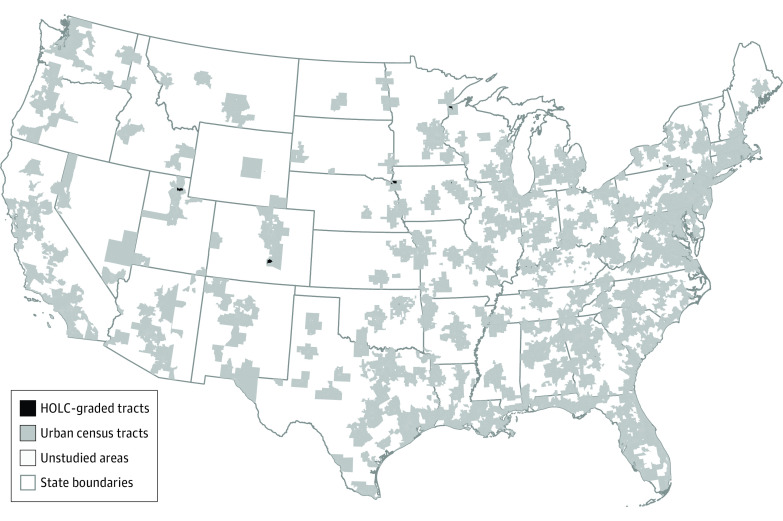
Map of Studied Areas A total of 59 231 urban census tracts and 15 011 Home Owners’ Loan Corporation (HOLC)-graded census tracts were analyzed in this study.

**Table 1.** Descriptive Statistics of Health Outcomes, Built Environment Indicators, and Census Tract Level Covariates

Characteristic	Sample size, No.	Data^a^
Census tract level		
Sleeping problems	59 239	36.88 (5.37)
Diabetes	59 239	10.92 (3.82)
Asthma	59 239	9.80 (1.62)
Dilapidated buildings^b^	57 186	0.25 (0.12)
Green space^b^	57 194	0.86 (0.15)
Crosswalks^b^	57 194	0.04 (0.05)
Non–single family homes^b^	57 194	0.30 (0.24)
Single-lane roads^b^	57 194	0.68 (0.15)
Median household income, $	58 995	70 734.17 (35 233.28)
College-educated residents^b^	59 222	0.33 (0.20)
Age, median (IQR), y	59 228	39.06 (33.90-43.70)
Female residents^b^	59 231	0.51 (0.04)
Insured residents^b^	59 162	0.91 (0.07)
Owner-occupied housing^b^	59 108	0.63 (0.24)
Vacant housing^b^	59 113	0.10 (0.09)
Single female head of household with children^b^	58 880	0.25 (0.20)
HOLC grade, No. (%)^c^		
A	15 011	425 (2.8)
B	15 011	1562 (10.4)
C	15 011	3847 (25.6)
D	15 011	2381 (15.9)
Other	15 011	6796 (45.3)
Race and ethnicity, No. (%)^d^		
American Indian or Alaska Native	273 733 933	1 160 595 (0.4)
Hispanic	273 733 933	53 321 345 (19.5)
Native Hawaiian or other Pacific Islander	273 733 933	462 259 (0.2)
Non-Hispanic Asian	273 733 933	17 166 370 (6.3)
Non-Hispanic Black	273 733 933	35 985 480 (13.2)
White	273 733 933	158 043 260 (57.7)

Abbreviation: HOLC, Home Owners Loan Corporation.

^a^ Unless otherwise indicated, data are expressed as mean (SD).

^b^ Presented as a proportion ranging from 0 to 1, with higher values indicating a higher prevalence of the characteristic in the census tract.

^c^ The numerator is the number of nonmissing census tracts and the denominator is the number of total census tracts. Grades are as follows: A, best; B, still desirable; C, definitely declining; D, hazardous.

^d^ The numerator is the aggregated population for each racial and ethnic group and the denominator is the total population across all studied areas.

All unadjusted models demonstrated statistically significant differences between predominantly White neighborhoods and the other neighborhoods (eAppendix 5 in Supplement 1). After adjusting for socioeconomic factors, the coefficients generally decreased but remained statistically significant. Compared with predominantly White neighborhoods, all other neighborhoods had more dilapidated buildings (Table 2 and Table 3). Neighborhoods with predominantly minoritized racial or ethnic groups other than Black residents had on average 6% (*P* < .001), unclassified neighborhoods had 2% (*P* < .001), and predominantly Black neighborhoods had less than 1% (*P* = .03) more dilapidated buildings compared with predominantly White neighborhoods.

**Table 2.** Hierarchical Linear Model Regression With State Random Intercepts^a^

Variable	Built environment indicator of interest, %									
	Dilapidated building		Green space		Crosswalks		Non–single family home		Single-lane roads	
	Estimate (95% CI)	*P* value	Estimate (95% CI)	*P* value	Estimate (95% CI)	*P* value	Estimate (95% CI)	*P* value	Estimate (95% CI)	*P* value
Intercept	0.19 (0.14 to 0.24)	<.001	0.85 (0.79 to 0.91)	<.001	0.12 (0.11 to 0.14)	<.001	0.71 (0.63 to 0.79)	<.001	0.80 (0.74 to 0.86)	<.001
Racial composition^b^										
Unclassified	0.02 (0.02 to 0.02)	<.001	−0.02 (−0.03 to −0.02)	<.001	0.01 (0.01 to 0.01)	<.001	0.04 (0.04 to 0.05)	<.001	−0.003 (−0.01 to −0.0002)	.04
Predominantly Black	0.00 (0.00 to 0.01)	.03	−0.02 (−0.02 to −0.01)	<.001	0.01 (0.00 to 0.01)	<.001	0.06 (0.06 to 0.07)	<.001	0.04 (0.03 to 0.04)	<.001
Predominantly minoritized racial or ethnic groups other than Black	0.06 (0.06 to 0.06)	<.001	−0.11 (−0.12 to −0.11)	<.001	0.02 (0.02 to 0.02)	<.001	0.17 (0.16 to 0.17)	<.001	−0.04 (−0.05 to −0.04)	<.001
Covariate										
Log (household income)	0.005 (0.0008 to 0.01)	.02	−0.02 (−0.02 to −0.01)	<.001	−0.005 (−0.01 to −0.003)	<.001	−0.01 (−0.01 to 0.00)	.13	−0.03 (−0.03 to −0.02)	<.001
College-educated residents, %	0.05 (0.04 to 0.06)	<.001	0.07 (0.06 to 0.08)	<.001	0.05 (0.05 to 0.05)	<.001	0.16 (0.14 to 0.17)	<.001	0.01 (0.00 to 0.02)	.03
Age	0.00002 (−0.0001 to 0.0002)	.81	0.0005 (0.0003 to 0.0006)	<.001	0.0003 (0.0003 to 0.0004)	<.001	0.002 (0.001 to 0.002)	<.001	−0.002 (−0.0023 to −0.002)	<.001
Female residents, %	0.07 (0.05 to 0.09)	<.001	0.18 (0.15 to 0.20)	<.001	−0.01 (−0.02 to −0.00)	.01	−0.29 (−0.33 to −0.25)	<.001	0.23 (0.20 to 0.26)	<.001
Insured, %	0.03 (0.01 to 0.05)	<.001	−0.08 (−0.10 to −0.05)	<.001	−0.0004 (−0.01 to 0.01)	.89	−0.01 (−0.04 to 0.02)	.70	−0.07 (−0.09 to −0.05)	<.001
Owner-occupied housing, %	−0.13 (−0.14 to −0.12)	<.001	0.22 (0.21 to 0.23)	<.001	−0.09 (−0.09 to −0.09)	<.001	−0.57 (−0.58 to −0.56)	<.001	0.34 (0.33 to 0.34)	<.001
Vacant housing, %	−0.07 (−0.08 to −0.06)	<.001	−0.16 (−0.17 to −0.15)	<.001	−0.01 (−0.01 to −0.01)	<.001	0.29 (0.27 to 0.31)	<.001	−0.12 (−0.13 to −0.10)	<.001
Single-family home, %	−0.01 (−0.02 to −0.01)	<.001	0.02 (0.01 to 0.03)	<.001	−0.01 (−0.01 to −0.00)	<.001	−0.06 (−0.07 to −0.05)	<.001	0.08 (0.07 to 0.09)	<.001

^a^ For each of the 5 built environment indicators of interest, we constructed a neighborhood topology model with only neighborhood classifications and a full model adding other socioeconomic covariates.

^b^ Reference category is predominantly White.

**Table 3.** Random Effect Sizes^a^

Random effect size	Built environment indicator of interest				
	Dilapidated	Green space	Crosswalks	Non–single family home	Single-lane roads
σ^2^	0.01	0.01	0.00	0.03	0.01
τ for State	0.00	0.00	0.00	0.01	0.00
Intraclass correlation coefficient^b^	0.29	0.27	0.23	0.25	0.13
No. of observations	56 812	56 818	56 818	56 818	56 818
Marginal *R*^2^/conditional *R*^2^	0.091/0.354	0.202/0.415	0.260/0.430	0.380/0.533	0.202/0.306

^a^ Includes all 50 states and Washington, DC (N = 51).

^b^ Shows the proportion of variance explained at the state level.

Also, compared with predominantly White neighborhoods, neighborhoods with predominantly minoritized racial or ethnic groups other than Black residents had on average 11% (*P* < .001) less green space; predominantly Black neighborhoods, 2% (*P* < .001) less green space; and unclassified neighborhoods, 2% (*P* < .001) less green space. Predominantly Black and unclassified neighborhoods had on average 2% (*P* < .001) and neighborhoods with predominantly minoritized racial or ethnic groups other than Black residents had on average 3% (*P* < .001) more crosswalks than neighborhoods with predominantly White residents.

Furthermore, neighborhoods with predominantly minoritized racial or ethnic groups other than Black residents had 17% (*P* < .001) more non–single family homes compared with predominantly White neighborhoods; predominantly Black neighborhoods, 6% (*P* < .001); and unclassified neighborhoods, 4% (*P* < .001). Additionally, neighborhoods with a predominantly Black population had on average 4% (*P* < .001) more single-lane roads, while neighborhoods with predominantly minoritized racial or ethnic groups other than Black residents had 4% (*P* < .001) fewer single-lane roads than predominantly White neighborhoods. About 29% of the variation in dilapidated buildings, 27% of the variation in green space, 23% of the variation in crosswalks, 25% of the variation in non–single family homes, and 13% of the variation in single-lane roads in neighborhoods was at the state level.

### Mediation Analysis

Neighborhoods with predominantly Black residents, predominantly minoritized racial or ethnic groups other than Black residents, and unclassified populations were more likely to have worse sleeping quality and higher rates of diabetes compared with predominantly White neighborhoods (Table 4). Neighborhoods with predominantly Black residents had the highest rates of sleeping problems (total effect size, 7.66% [95% CI, 7.57%-7.75%]; *P* < .001) and diabetes (total effect size, 4.95% [95% CI, 4.88%-5.03%]; *P* < .001). This was followed by neighborhoods with predominantly minoritized racial or ethnic groups other than Black for total effect size of sleeping problems (1.44% [95% CI, 1.34%-1.53%]; *P* < .001) and total effect size of diabetes (2.79% [2.71%-2.87%]; *P* < .001]) and unclassified neighborhoods for total effect size of sleeping problems (1.89% [95% CI, 1.84%-1.94%]; *P* < .001) and total effect size of diabetes (1.26% [95% CI, 1.22%-1.30%]; *P* < .001) when compared with predominantly White neighborhoods. For asthma, only neighborhoods with predominantly Black residents had a higher prevalence than predominantly White neighborhoods (total effect size, 1.40% [95% CI, 1.37%-1.43%]; *P* < .001).

**Table 4.** Estimates of Total Effect Sizes of the Association Between Neighborhood Topology and Health Outcomes Mediated by Built Environment Indicators

Health outcomes	Built environment indicators, estimated total effect size, % (95% CI)				
	Dilapidated building	Green space	Crosswalks	Non–single family home	Single-lane roads
Black vs White neighborhoods					
Sleeping problems	7.66 (7.57 to 7.75)	7.66 (7.57 to 7.75)	7.66 (7.57 to 7.75)	7.66 (7.57 to 7.75)	7.66 (7.57 to 7.75)
Diabetes	4.95 (4.88 to 5.03)	4.87 (4.88 to 5.03)	4.95 (4.88 to 5.03)	4.95 (4.88 to 5.04)	4.95 (4.88 to 5.03)
Asthma	1.40 (1.38 to 1.43)	1.40 (1.37 to 1.43)	1.40 (1.37 to 1.43)	1.40 (1.37 to 1.43)	1.40 (1.37 to 1.43)
Predominantly minoritized racial or ethnic group neighborhoods other than Black vs White neighborhoods					
Sleeping problems	1.44 (1.34 to 1.53)	1.44 (1.35 to 1.52)	1.44 (1.35 to 1.52)	1.44 (1.35 to 1.53)	1.44 (1.35 to 1.52)
Diabetes	2.79 (2.71 to 2.86)	2.79 (2.72 to 2.86)	2.79 (2.71 to 2.86)	2.79 (2.72 to 2.87)	2.79 (2.72 to 2.86)
Asthma	−1.08 (−1.11 to −1.05)	−1.08 (−1.11 to −1.05)	−1.08 (−1.11 to −1.05)	−1.10 (−1.11 to −1.05)	−1.08 (−1.11 to −1.05)
Unclassified vs White neighborhoods					
Sleeping problems	1.89 (1.84 to 1.94)	1.89 (1.83 to 1.94)	1.89 (1.84 to 1.94)	1.89 (1.85 to 1.53)	1.89 (1.84 to 1.94)
Diabetes	1.26 (1.22 to 1.30)	1.26 (1.22 to 1 30)	1.26 (1.22 to 1.30)	1.26 (1.22 to 1.30)	1.26 (1.22 to 1.30)
Asthma	−0.10 (−0.11 to −0.08)	−0.10 (−0.11 to −0.08)	−0.10 (−0.11 to −0.08	−0.10 (−0.11 to −0.80)	−0.10 (−0.11 to −0.08)

Also, all built environment indicators significantly mediated the association between neighborhood racial and ethnic composition and health outcomes (eAppendix 6 in Supplement 1). The most significant mediator was non–single family homes, which mediated the association between neighborhoods with predominantly minoritized racial or ethnic groups other than Black residents and sleeping problems by 12.8% and the association between unclassified neighborhoods and asthma by 24.2%. After controlling for socioeconomic covariates, the mediation of the associations between neighborhood composition and health outcomes was reduced but was mostly still significant.

### HOLC Grades

A χ^2^ test indicated an association between HOLC grades and neighborhood classifications by racial and ethnic groups (eAppendix 7 in Supplement 1). Compared with grade A census tracts, the other grades had more dilapidated buildings, less green space, more crosswalks, more non–single family homes, and fewer single-lane roads. After adjusting for social and economic conditions, grades B and C census tracts had on average 2% (*P* = .004 and *P* < .001, respectively) and grade D tracts had on average 3% (*P* < .001) more dilapidated buildings. For green space, grade B census tracts had on average 2% (*P* < .001) less green space than grade A census tracts; grade C census tracts, 5% (*P* < .001) less green space; and grade D census tracts, 11% (*P* < .001) less green space. The disparities for crosswalks between tracts with different grades were significant, though smaller; grade B and C tracts had 1% (*P* < .001) and grade D tracts had 2% (*P* < .001) more crosswalks compared with grade A tracts. Tracts classified as grades B and D had 7% (*P* < .001) and 18% (*P* < .001) more non–single family homes, respectively, compared with grade A tracts. For single-lane roads, only grades C and D tracts had significant coefficients, with 2% (*P* = .002) and 8% (*P* < .001) fewer single-lane roads, respectively, compared with grade A tracts.

## Discussion

Built environment data extracted from online street-level images could provide insights into large-scale patterns of inequality in the built environment and its association with neighborhood racial and ethnic group composition and health outcomes. Predominantly White neighborhoods or neighborhoods with HOLC grade A had more built environment resources that have been associated with good health compared with neighborhoods with predominantly Black residents, members of other racial or ethnic minoritized populations, and predominantly unclassified residents. For example, neighborhood walkability, urban development, and green space are associated with improved physical and mental health.^19^

While our findings about the prevalence of dilapidated buildings^42^ and access to greenery^43^ are not novel, the scale is larger, expanding findings that have mostly been reported at a local level to a national scale. Our use of this novel data source highlights new methods for scalable built environment assessment and reveals the need for collection of quality built environment data that could be addressed by policy. Building accessible, comprehensive, and easy-to-use data platforms could substantially improve monitoring of social determinants of health and further contribute to closing racial and ethnic health inequities.^44^

We observed that neighborhoods with predominantly Black residents, predominantly members of other racial or ethnic minoritized populations, and unclassified residents had more crosswalks compared with predominantly White neighborhoods, a finding reported in other studies.^45,46,47^ Thornton et al^47^ posited that neighborhoods with predominantly racial or ethnic minoritized populations are usually located in older areas of cities that were developed to be more pedestrian friendly. However, additional studies are needed to understand the reasons for these differences so that appropriate policy solutions can be adopted. Also, non–single family homes were less prevalent in White neighborhoods, which we found to be associated with homeownership (eAppendix 4 in Supplement 1). There are many studies^48,49^ showing that Black individuals and members of other racial and ethnic minority groups have less homeownership when compared with White individuals, and these disparities have been associated with poor health outcomes.^50^

Built environment as a social determinant of health has been associated with structural racism that operates through policies that distribute burdens and benefits unfairly to people and neighborhoods based on race.^51,52,53^ While the magnitude of the differences among the other 3 neighborhood classifications and White neighborhoods varied, especially after controlling for socioeconomic conditions, it is important to note that the intersection of structural racism and social, economic, and demographic factors increases disadvantage for some groups.^54,55^ In contrast, structural racism benefits White individuals and neighborhoods with higher proportions of White residents by providing access to built environment resources that promote health based on race. Predominately White neighborhoods had fewer dilapidated buildings, more green space, fewer non–single family homes, and more single-lane roads. Studies have shown that slavery and New Deal policies benefited White individuals by expanding their economic advantage.^56,57^

Furthermore, except for the single-lane road model, between 20% and 29% of the variance in our models was at the state level, suggesting some percentage of deviations in neighborhood built environment characteristics are associated with state-level differences. State-level policies have been shown to influence the inequitable distribution of social and economic resources that influence differences in health outcomes across racial and ethnic groups and neighborhoods.^58,59^ Policies such as the Community Reinvestment Act and Neighborhood Homes Investment Act have the potential to improve built environment resources.^60,61^ However, researchers recommend that such policies should explicitly address race and ethnicity to tackle disparities and should ensure that residents and businesses are not priced out of their neighborhoods.^62^

### Limitations

There are some limitations to this study. First, the data set was collected per image availability. Since these images are part of a commercial service and their availability generally leans toward the places that have a larger market, the available number of images might have spatial variability. This might explain the lack of associations between predominantly Black neighborhoods and the built environment features, which is contrary to previous studies.^43,63,64,65^ Second, both street-level image indicators and PLACES variables are model estimates, and the data quality hinges on the model performance. Third, we recognize the need for strong assumptions of causal mediation analysis, and due to the heterogeneous nature of the data sources, these assumptions might not be strictly met; thus, causal interpretations should be avoided. Fourth, there is a need for an intersectional approach to assess how overlapping socioeconomic disadvantage, racism, sexism, and other systems of oppression interact to shape health.^54^ Fifth, studies on neighborhoods have used varied definitions, including census tracts, zip codes, census block groups, and local administrative neighborhoods. However, these definitions of neighborhoods are not necessarily synonymous with neighborhood delineations defined by communities.^54^ Additionally, our selected covariates are not comprehensive; there are other factors that interact with the various indicators.

## Conclusions

In this cross-sectional study of built environment indicators derived from street-level images, predominantly White neighborhoods were generally associated with better built environment conditions such as more greenery, fewer dilapidated buildings, and fewer non–single family homes. These built environment variables also partially mediated the association between neighborhood racial and ethnic composition and health outcomes. Improvements in data quality on a national scale would provide deeper insights into the association among health outcomes, built environment features, and neighborhoods.

## Notes

Supplement 1. **eAppendix 1.** Cluster Analysis **eAppendix 2.** Mediation Model Framework **eAppendix 3.** Built Environment Data Processing **eAppendix 4.** Distribution of Health Outcomes and Built Environment Indicators **eAppendix 5.** Additional Details on Statistical Analysis **eAppendix 6.** Mediation Analysis **eAppendix 7.** HOLC Analysis **eAppendix 8.** Neighborhood and Sensitivity Analysis
eReferences

Supplement 2.
Data Sharing Statement
