# Supplementary material for: Association of Neighborhood Racial and Ethnic Composition and Historical Redlining With Built Environment Indicators Derived From Street View Images in the US
Source: JAMA Netw Open. 2023 Jan 18;6(1):e2251201. doi: 10.1001/jamanetworkopen.2022.51201 (PMC9856713; doi:10.1001/jamanetworkopen.2022.51201)
Supplement: Supplement 1. — eAppendix 1. Cluster Analysis eAppendix 2. Mediation Model Framework eAppendix 3. Built Environment Data Processing eAppendix 4. Distribution of Health Outcomes and Built Environment Indicators eAppendix 5. Additional Details on Statistical Analysis eAppendix 6. Mediation Analysis eAppendix 7. HOLC Analysis eAppendix 8. Neighborhood and Sensitivity Analysis eReferences [file jamanetwopen-e2251201-s001.pdf]

Supplementary Online Content

Yang Y, Cho A, Nguyen Q, Nsoesie EO. Association of neighborhood racial and ethnic composition and historical redlining with built environment indicators derived from street-view images in the US. *JAMA Netw Open*. 2023;6(1):e2251201. doi:10.1001/jamanetworkopen.2022.51201

eAppendix 1. Cluster Analysis
eAppendix 2. Mediation Model Framework
eAppendix 3. Built Environment Data Processing
eAppendix 4. Distribution of Health Outcomes and Built Environment Indicators
eAppendix 5. Additional Details on Statistical Analysis
eAppendix 6. Mediation Analysis
eAppendix 7. HOLC Analysis
eAppendix 8. Neighborhood and Sensitivity Analysis
eReferences

This supplementary material has been provided by the authors to give readers additional information about their work.

© 2023 Yang Y et al. *JAMA Network Open*.

eAppendix 1. Cluster Analysis

We applied clustering methods to assess potential groupings between percent race/ethnicity in a neighborhood and built environment variables. We used clustering large applications (CLARA), an extension of k-medoids clustering technique.[1] CLARA is a sampling and partition-based clustering method typically used for analyzing large datasets. It uses the Manhattan distance and inherits k-medoids' robustness to outliers and noise.[2]

First, we determined the optimal number of clusters (K) using the gap statistics metrics, which is a commonly used method for evaluating the quality of clustering.[3] The optimum K was 4 and the clusters were as follows: 19,069 (40.8%) census tract in the first cluster, 15,541 (33.2%) in the second, 8,297 (17.8%) in the third and 3,835 (8.2%) in the fourth.

Figure S1A shows the distribution of built environment indicators across the four clusters. Cluster 1 represents the census tracts that had relatively more green space, but fewer crosswalks, non-single family homes, and dilapidated buildings. Cluster 1 tracts also had more single lane roads compared to others. Conversely, cluster 3 and 4 represent tracts with relatively more dilapidated buildings, crosswalks, and non-single family homes, but with less green space and fewer single lanes. This trend was most pronounced in cluster 4. Cluster 2 captured census tracts that did not fall into the extremes—the majority of the tracts had relatively moderate to high values for all built environment indicators; the peak of the distribution was around 50% percentile for all built environment indicators.

© 2023 Yang Y et al. *JAMA Network Open*.

eFigure 1A. Distribution of Built Environment Indicators (in Percentile) by Cluster

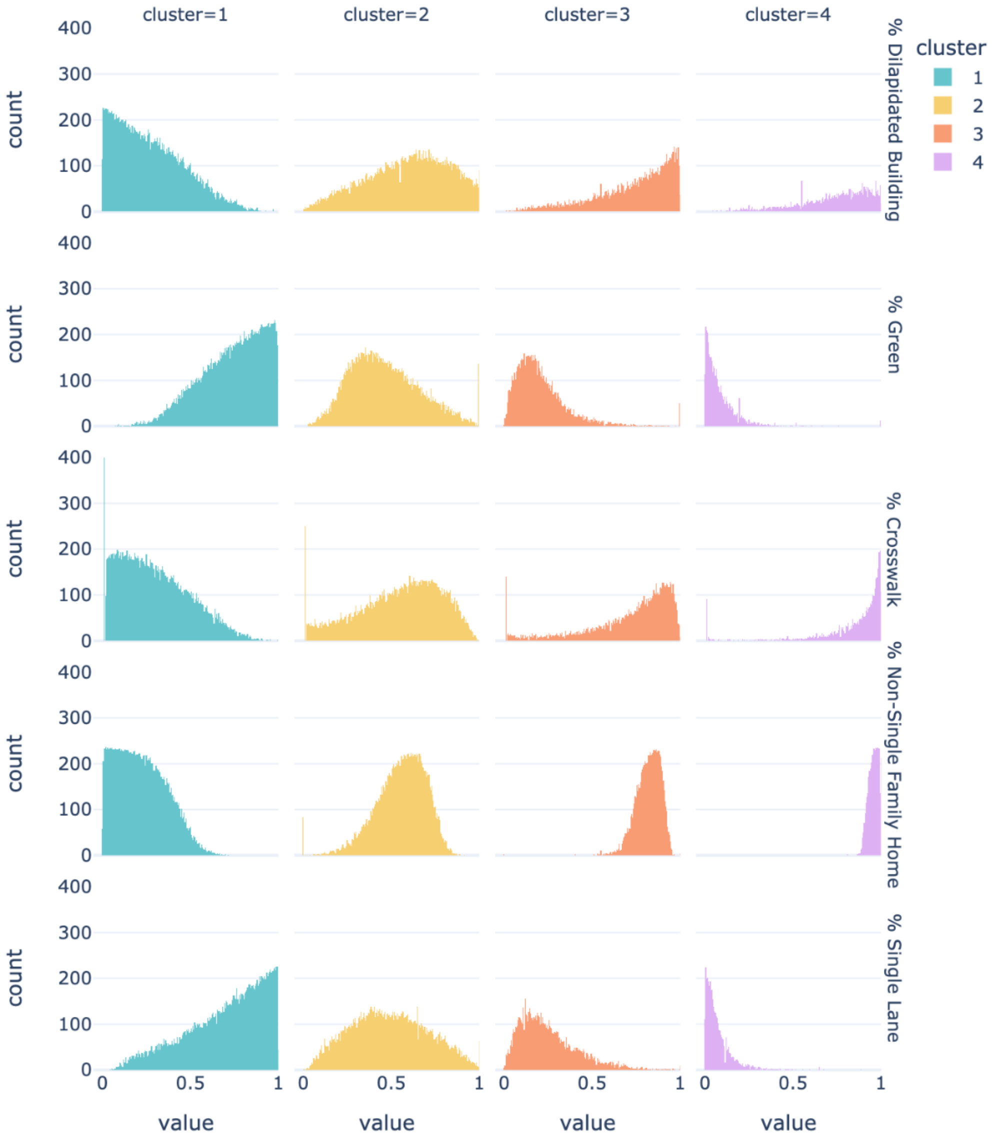

Figure S1B shows the distribution of the percent of the population representing each race/ethnicity normalized by the percentile. The percentage of White residents in the census tract represented in each cluster decreased as we move from cluster one to cluster four. The percentage of Hispanic residents in the census tracts appeared to increase from cluster one through four. Compared to percent of White residents and percent of Hispanic residents in the tract, the percent of other racial and ethnic groups in the census tracts were not strongly represented across the clusters.

© 2023 Yang Y et al. *JAMA Network Open*.

Based on Figures S1A and S1B, we noted that neighborhoods with a higher percentage of White residents tend to have better built environment indicators compared to others.

eFigure 1B. Distribution of Percent Race/Ethnic Population Across Clusters

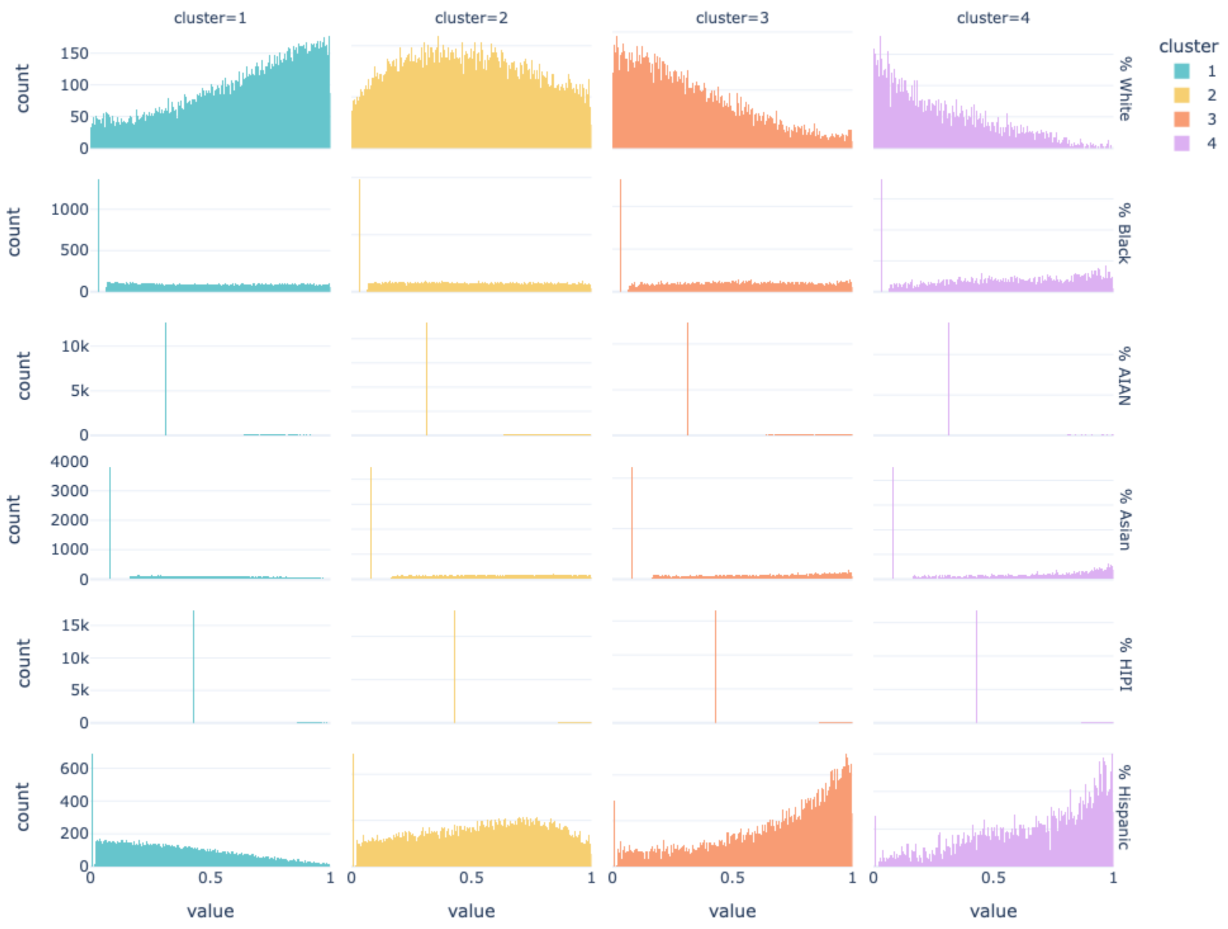

© 2023 Yang Y et al. *JAMA Network Open*.

eAppendix 2. Mediation Model Framework

To study how structural racism in the built environment indicators mediate the relationship between neighborhood racial composition and adverse health outcomes, we constructed a series of multilevel mediation models. Figure S2 illustrates our model framework.

eFigure 2. Mediation Model Framework

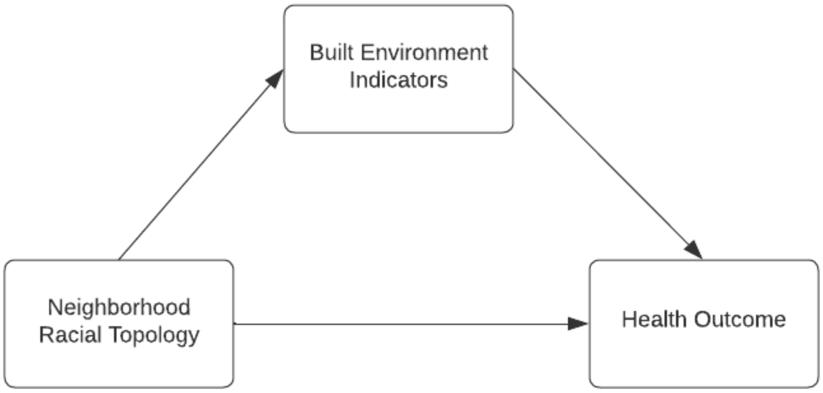

© 2023 Yang Y et al. *JAMA Network Open*.

eAppendix 3. Built Environment Data Processing

Model Training

We used data described in Nguyen et al.[4–6] The data consisted of 164 million images extracted from Google Street Views (GSV) Application Programming Interface (API) in November 2019. Convolutional Neural Networks (CNNs) - the state-of-art model for computer vision tasks - were used to identify objects in the collected images. To implement the object identification task, three different training sets were created. For the first dataset, which consisted of street greenness, crosswalk, single-lane road, building type, and visible wire, Nguyen et al. manually annotated 18,700 images from Chicago, Illinois; Salt Lake City, UT; Charleston, West Virginia; and a national sample. The model was trained on a standard deep CNN network architecture - Visual Geometry Group VGG-19 - in TensorFlow with sigmoid cross-entropy and logits as the loss function. For the second dataset focused on dilapidated buildings, 59,000 images were downloaded from Flickr with the keywords "old or dilapidated building" and "nice building". A pre-trained ResNet18 network[19] trained on the ImageNet dataset was used. The last fully connected layer was replaced with two fully connected layers that separately predicted labels for GSV images and Flickr images. Lastly, for sidewalks, the training set was constructed by annotating 24,300 GSV images from New Jersey. For other indicators, such as street lights, two or more cars, street signs, and chain-linked fences, Nguyen et al. sampled and annotated 18,000 images from the national collection of GSV images.[23] All models were trained and hyper-tuned by splitting the data into a training set and validation set using an 80:20 ratio for best model performance.

Model Validation

Accuracies were estimated from the test set and were above 82% for all examined built environment indicators. Accuracies were the following for the various neighborhood built environment indicators: street lights (88%); two or more cars (88%); street signs (82%); chain-link fence (95%), green space (88.70%), presence of crosswalks (97.20%), non-single family homes (82.35%), single lane roads (88.41%), visible utility wires (83.00%), sidewalks 84.5%, and dilapidated buildings (93.4%). Additional information on the methods can be found in Nguyen et al.[6]

Indicators

To produce neighborhood-level indicators, the binary indicators of each image were aggregated at the census tract level. The aggregated data indicated the percentage of all GSV images in the tract that contained these built environment elements. The resulting dataset consisted of eleven built environment indicators: dilapidated buildings, two or more cars, chain link, street signs, street lights, green spaces, crosswalks, non-single family home, single lane roads, visible wire, and sidewalk. The GSV dataset covered 72,311 census tracts across the U.S.

© 2023 Yang Y et al. *JAMA Network Open*.

eAppendix 4. Distribution of Health Outcomes and Built Environment Indicators

eFigure 3 illustrates the distribution of health outcomes and built environment indicators. The distribution of the built environment indicators was highly skewed, especially for green space and crosswalks, the values were generally high and low, respectively. For the health outcome variables, the spread and the center of the distributions were generally low, implying a low variation. We observed differences in the distributions for different neighborhood racial topologies, for which the contrast was most obvious between predominantly White tracts and predominantly minoritized racial group other than Black tracts.

eFigure 3. Distribution of Built Environment Indicators and Health Outcomes Faceted by Neighborhood Topology

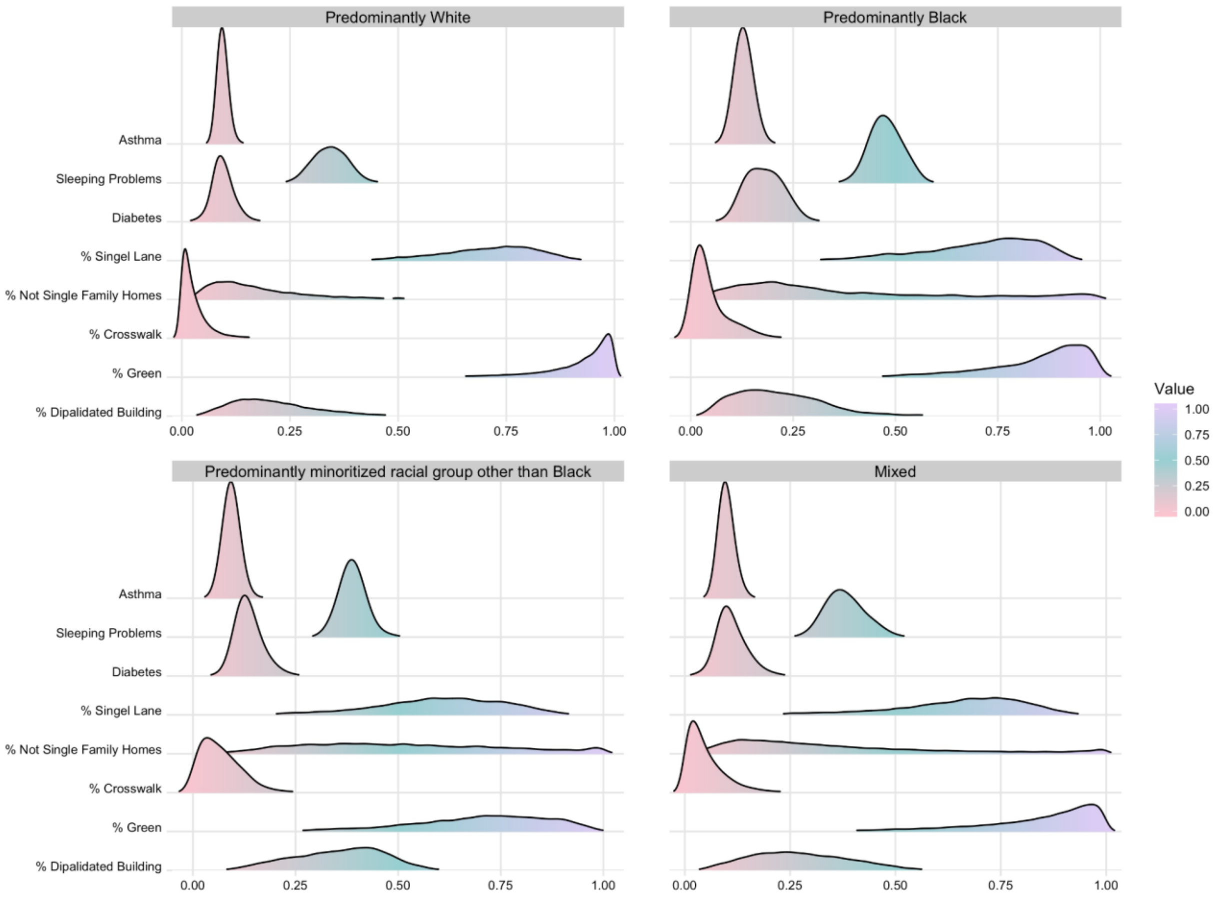

eFigure 4 and eTable 1 show the results of our preliminary correlation analysis. We observed differences in the associations between the built environment characteristics and the percentage of different racial/ethnic groups in a neighborhood. The percent of White residents was negatively correlated with four out of five of the built environment indicators: dilapidated buildings (r = -0.29), non-single family homes (r = -0.41), and crosswalks (r = -0.34), but positively correlated with green space (r = 0.36) and single lanes (r = 0.14). In contrast, the percent of Black residents and other minoritized populations were negatively associated with green space, and positively correlated

with dilapidated buildings, crosswalks, and single lanes. These associations were strongest for percent Hispanic residents; these neighborhoods had less green space (*r*= -0.40) and more dilapidated buildings (*r* = 0.36). Furthermore, the magnitude of the correlation was stronger for percent Asian and Hispanics, compared to percent Black residents in a census tract. All correlations between percent Black residents and built environment indicators were less than 0.3.

eFigure 4. Correlation Heatmap for Urban Tracts

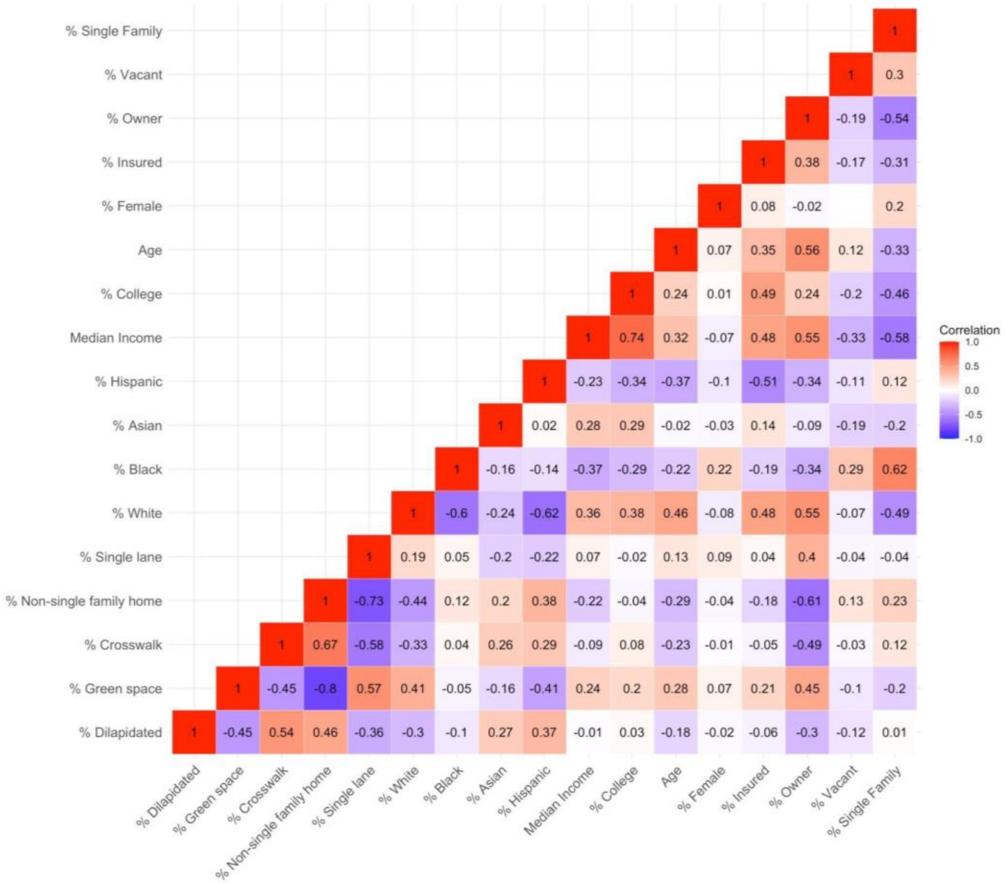

Only statistically significant associations are shown with text labels.

© 2023 Yang Y et al. *JAMA Network Open*.

eTable 1. Correlation Coefficients With 95% CIs

Corr is the correlation coefficient. *N* is the sample size. *CI* is the half-width confidence interval. *LL* and *UL* are the Lower Limit and the Upper Limit of the full-width confidence interval. The calculation was done using the *metan*[7] package in R.

Variable 1	*Variable 2*	*Corr*	*N*	*CI*	*LL*	*UL*
% Dilapidated	% Green space	-0.46	56810	0.01	-0.47	-0.45
% Dilapidated	% Crosswalk	0.55	56810	0.01	0.54	0.55
% Dilapidated	% Non-single Family Home	0.47	56810	0.01	0.46	0.47
% Dilapidated	% Single Lane	-0.36	56810	0.01	-0.37	-0.35
% Dilapidated	% Non-Hispanic White	-0.30	56810	0.01	-0.31	-0.29
% Dilapidated	% Non-Hispanic Black	-0.11	56810	0.01	-0.11	-0.10
% Dilapidated	% Non-Hispanic Asian	0.27	56810	0.01	0.26	0.28
% Dilapidated	% Hispanic	0.37	56810	0.01	0.36	0.38
% Dilapidated	Median Household Income	-0.00	56810	0.01	-0.01	0.01
% Dilapidated	% College	0.04	56810	0.01	0.03	0.05
% Dilapidated	Age	-0.18	56810	0.01	-0.19	-0.17
% Dilapidated	% Female	-0.02	56810	0.01	-0.03	-0.01
% Dilapidated	% Insured	-0.05	56810	0.01	-0.06	-0.05
% Dilapidated	% Owner Occupied Housing	-0.31	56810	0.01	-0.31	-0.30
% Dilapidated	% Vacant Housing	-0.12	56810	0.01	-0.13	-0.11
% Dilapidated	% Single Female Head of Household with Children	0.01	56810	0.01	0.01	0.02
% Green space	% Crosswalk	-0.44	56810	0.01	-0.45	-0.44
% Green space	% Non-single Family Home	-0.79	56810	0.00	-0.80	-0.79
% Green space	% Single Lane	0.55	56810	0.01	0.55	0.56
% Green space	% Non-Hispanic White	0.40	56810	0.01	0.39	0.40
% Green space	% Non-Hispanic Black	-0.04	56810	0.01	-0.04	-0.03
% Green space	% Non-Hispanic Asian	-0.17	56810	0.01	-0.17	-0.16
% Green space	% Hispanic	-0.41	56810	0.01	-0.42	-0.40
% Green space	Median Household Income	0.22	56810	0.01	0.21	0.23
% Green space	% College	0.19	56810	0.01	0.18	0.20
% Green space	Age	0.28	56810	0.01	0.27	0.28
% Green space	% Female	0.08	56810	0.01	0.07	0.09
% Green space	% Insured	0.21	56810	0.01	0.20	0.22
% Green space	% Owner Occupied Housing	0.43	56810	0.01	0.42	0.44
% Green space	% Vacant Housing	-0.08	56810	0.01	-0.09	-0.07
% Green space	% Single Female Head of Household with Children	-0.19	56810	0.01	-0.19	-0.18
% Crosswalk	% Non-single Family Home	0.67	56810	0.01	0.67	0.68
% Crosswalk	% Single Lane	-0.58	56810	0.01	-0.59	-0.58
% Crosswalk	% Non-Hispanic White	-0.32	56810	0.01	-0.33	-0.32
% Crosswalk	% Non-Hispanic Black	0.04	56810	0.01	0.03	0.05
% Crosswalk	% Non-Hispanic Asian	0.26	56810	0.01	0.25	0.27
% Crosswalk	% Hispanic	0.27	56810	0.01	0.26	0.28
% Crosswalk	Median Household Income	-0.08	56810	0.01	-0.09	-0.07
% Crosswalk	% College	0.10	56810	0.01	0.09	0.11
% Crosswalk	Age	-0.22	56810	0.01	-0.23	-0.22
% Crosswalk	% Female	-0.02	56810	0.01	-0.03	-0.01
% Crosswalk	% Insured	-0.05	56810	0.01	-0.06	-0.04
% Crosswalk	% Owner Occupied Housing	-0.50	56810	0.01	-0.50	-0.49
% Crosswalk	% Vacant Housing	-0.03	56810	0.01	-0.04	-0.02

© 2023 Yang Y et al. *JAMA Network Open*.

% Crosswalk	% Single Female Head of Household with Children	0.13	56810	0.01	0.12	0.13
% Non-single Family Home	% Single Lane	-0.73	56810	0.01	-0.74	-0.73
% Non-single Family Home	% Non-Hispanic White	-0.43	56810	0.01	-0.44	-0.43
% Non-single Family Home	% Non-Hispanic Black	0.11	56810	0.01	0.10	0.12
% Non-single Family Home	% Non-Hispanic Asian	0.21	56810	0.01	0.20	0.21
% Non-single Family Home	% Hispanic	0.37	56810	0.01	0.36	0.38
% Non-single Family Home	Median Household Income	-0.21	56810	0.01	-0.21	-0.20
% Non-single Family Home	% College	-0.03	56810	0.01	-0.04	-0.02
% Non-single Family Home	Age	-0.28	56810	0.01	-0.29	-0.28
% Non-single Family Home	% Female	-0.06	56810	0.01	-0.07	-0.05
% Non-single Family Home	% Insured	-0.18	56810	0.01	-0.19	-0.17
% Non-single Family Home	% Owner Occupied Housing	-0.60	56810	0.01	-0.61	-0.60
% Non-single Family Home	% Vacant Housing	0.12	56810	0.01	0.12	0.13
% Non-single Family Home	% Single Female Head of Household with Children	0.23	56810	0.01	0.22	0.23
% Single Lane	% Non-Hispanic White	0.18	56810	0.01	0.17	0.19
% Single Lane	% Non-Hispanic Black	0.06	56810	0.01	0.05	0.07
% Single Lane	% Non-Hispanic Asian	-0.20	56810	0.01	-0.21	-0.19
% Single Lane	% Hispanic	-0.20	56810	0.01	-0.21	-0.19
% Single Lane	Median Household Income	0.07	56810	0.01	0.06	0.07
% Single Lane	% College	-0.04	56810	0.01	-0.05	-0.03
% Single Lane	Age	0.13	56810	0.01	0.13	0.14
% Single Lane	% Female	0.10	56810	0.01	0.09	0.11
% Single Lane	% Insured	0.04	56810	0.01	0.03	0.05
% Single Lane	% Owner Occupied Housing	0.40	56810	0.01	0.40	0.41
% Single Lane	% Vacant Housing	-0.04	56810	0.01	-0.05	-0.03
% Single Lane	% Single Female Head of Household with Children	-0.04	56810	0.01	-0.05	-0.03
% Non-Hispanic White	% Non-Hispanic Black	-0.59	56810	0.01	-0.59	-0.58
% Non-Hispanic White	% Non-Hispanic Asian	-0.24	56810	0.01	-0.25	-0.24
% Non-Hispanic White	% Hispanic	-0.63	56810	0.01	-0.64	-0.63
% Non-Hispanic White	Median Household Income	0.35	56810	0.01	0.35	0.36
% Non-Hispanic White	% College	0.37	56810	0.01	0.37	0.38
% Non-Hispanic White	Age	0.46	56810	0.01	0.45	0.46
% Non-Hispanic White	% Female	-0.07	56810	0.01	-0.08	-0.06
% Non-Hispanic White	% Insured	0.50	56810	0.01	0.49	0.50
% Non-Hispanic White	% Owner Occupied Housing	0.54	56810	0.01	0.53	0.55
% Non-Hispanic White	% Vacant Housing	-0.05	56810	0.01	-0.06	-0.04
% Non-Hispanic White	% Single Female Head of Household with Children	-0.48	56810	0.01	-0.49	-0.48
% Non-Hispanic Black	% Non-Hispanic Asian	-0.16	56810	0.01	-0.16	-0.15

© 2023 Yang Y et al. *JAMA Network Open*.

% Non-Hispanic Black	% Hispanic	-0.14	56810	0.01	-0.15	-0.13
% Non-Hispanic Black	Median Household Income	-0.37	56810	0.01	-0.38	-0.36
% Non-Hispanic Black	% College	-0.29	56810	0.01	-0.30	-0.29
% Non-Hispanic Black	Age	-0.22	56810	0.01	-0.22	-0.21
% Non-Hispanic Black	% Female	0.22	56810	0.01	0.21	0.22
% Non-Hispanic Black	% Insured	-0.19	56810	0.01	-0.20	-0.18
% Non-Hispanic Black	% Owner Occupied Housing	-0.34	56810	0.01	-0.35	-0.33
% Non-Hispanic Black	% Vacant Housing	0.26	56810	0.01	0.26	0.27
% Non-Hispanic Black	% Single Female Head of Household with Children	0.61	56810	0.01	0.60	0.61
% Non-Hispanic Asian	% Hispanic	0.01	56810	0.01	-0.00	0.02
% Non-Hispanic Asian	Median Household Income	0.29	56810	0.01	0.28	0.29
% Non-Hispanic Asian	% College	0.31	56810	0.01	0.30	0.31
% Non-Hispanic Asian	Age	-0.03	56810	0.01	-0.04	-0.02
% Non-Hispanic Asian	% Female	-0.03	56810	0.01	-0.04	-0.02
% Non-Hispanic Asian	% Insured	0.14	56810	0.01	0.13	0.15
% Non-Hispanic Asian	% Owner Occupied Housing	-0.10	56810	0.01	-0.11	-0.09
% Non-Hispanic Asian	% Vacant Housing	-0.18	56810	0.01	-0.19	-0.18
% Non-Hispanic Asian	% Single Female Head of Household with Children	-0.19	56810	0.01	-0.20	-0.18
% Hispanic	Median Household Income	-0.23	56810	0.01	-0.24	-0.23
% Hispanic	% College	-0.35	56810	0.01	-0.35	-0.34
% Hispanic	Age	-0.36	56810	0.01	-0.37	-0.36
% Hispanic	% Female	-0.10	56810	0.01	-0.11	-0.09
% Hispanic	% Insured	-0.54	56810	0.01	-0.55	-0.53
% Hispanic	% Owner Occupied Housing	-0.32	56810	0.01	-0.33	-0.32
% Hispanic	% Vacant Housing	-0.11	56810	0.01	-0.11	-0.10
% Hispanic	% Single Female Head of Household with Children	0.12	56810	0.01	0.11	0.13
Median Household Income	% College	0.74	56810	0.01	0.74	0.75
Median Household Income	Age	0.30	56810	0.01	0.29	0.31
Median Household Income	% Female	-0.07	56810	0.01	-0.08	-0.06
Median Household Income	% Insured	0.49	56810	0.01	0.49	0.50
Median Household Income	% Owner Occupied Housing	0.54	56810	0.01	0.53	0.55
Median Household Income	% Vacant Housing	-0.32	56810	0.01	-0.33	-0.31
Median Household Income	% Single Female Head of Household with Children	-0.58	56810	0.01	-0.59	-0.58
% College	Age	0.22	56810	0.01	0.21	0.23
% College	% Female	0.01	56810	0.01	0.01	0.02
% College	% Insured	0.50	56810	0.01	0.49	0.51
% College	% Owner Occupied Housing	0.22	56810	0.01	0.21	0.23
% College	% Vacant Housing	-0.19	56810	0.01	-0.20	-0.18
% College	% Single Female Head of Household with Children	-0.46	56810	0.01	-0.46	-0.45
Age	% Female	0.08	56810	0.01	0.07	0.09
Age	% Insured	0.35	56810	0.01	0.34	0.35
Age	% Owner Occupied Housing	0.55	56810	0.01	0.54	0.55
Age	% Vacant Housing	0.14	56810	0.01	0.14	0.15

© 2023 Yang Y et al. *JAMA Network Open*.

Age	% Single Female Head of Household with Children	-0.31	56810	0.01	-0.32	-0.30
% Female	% Insured	0.09	56810	0.01	0.08	0.10
% Female	% Owner Occupied Housing	-0.01	56810	0.01	-0.02	-0.00
% Female	% Vacant Housing	-0.01	56810	0.01	-0.01	0.00
% Female	% Single Female Head of Household with Children	0.19	56810	0.01	0.18	0.20
% Insured	% Owner Occupied Housing	0.39	56810	0.01	0.39	0.40
% Insured	% Vacant Housing	-0.17	56810	0.01	-0.17	-0.16
% Insured	% Single Female Head of Household with Children	-0.32	56810	0.01	-0.33	-0.31
% Owner Occupied Housing	% Vacant Housing	-0.17	56810	0.01	-0.18	-0.16
% Owner Occupied Housing	% Single Female Head of Household with Children	-0.53	56810	0.01	-0.54	-0.53
% Vacant Housing	% Single Female Head of Household with Children	0.28	56810	0.01	0.27	0.29

© 2023 Yang Y et al. *JAMA Network Open*.

eAppendix 5. Additional Details on Statistical Analysis

All statistical analysis was conducted in R using the lme4[8], cluster[9], and mediation[10] packages and statistical significance was set to α=0.05. We first conducted correlation and cluster analyses to examine the association between the built environment and neighborhood percent race/ethnicity (described in Appendix 1 and Appendix 4). Next, we performed a multilevel linear regression analysis using the built environment indicators as the dependent variables and neighborhood racial topology as the independent variable. We used the predominantly White neighborhood classification as the reference group. The reason for using multilevel regression was to account for the clustering that might happen at the census tract level; the baseline level of the value for our variables might differ from state to state. Therefore, in our hierarchical linear model, we included a random intercept for state to account for potential correlation in census tracts within the same state. We considered county-level random intercepts but did not include it in our study because of data sparsity which increases the risk of overestimation of group level variances.[11] We used the socioeconomic indicators as the covariates.

To study how the influence of structural racism in the built environment indicators mediates the relationship between neighborhood racial composition and adverse health outcomes, we constructed a series of multilevel mediation models. The mediation was conducted using a model-based approach.[10] First, we fit a mediator model where the potential mediator - built environment - was estimated by our treatment variable - the neighborhood racial composition. Then, we constructed the model for our outcome variable - separately for each of the health outcome variables - where the independent variables were neighborhood racial composition and the built environment. Further, we obtained the estimated average causal mediation effects, total effects, direct effects, proportion mediated, and other statistics of interest. We then determined the type of mediation of the built environment indicators: partial mediation, full mediation, or no mediation. Each model was fit using a hierarchical linear model adjusting for the same covariates and a random intercept for different states, consistent with our previous multilevel analysis.

Since our treatment variable, neighborhood racial topology, was categorical, in our mediation model, we constructed a contrast between predominantly White neighborhoods and the other three types of neighborhoods. Thus, for each of the built environment variables, we developed three models assessing whether the built environment indicators mediated the association between the difference in racial composition and health outcomes. The three types of neighborhood topology comparisons were: 1) predominantly Black referencing predominantly White, 2) predominantly minoritized racial group other than Black referencing predominantly White, and 3) unclassified neighborhoods referencing predominantly White. Each model included the same set of socioeconomic variables as covariates and a random intercept for state, consistent with our multilevel linear regression models.

© 2023 Yang Y et al. *JAMA Network Open*.

eTable 2. HLM Models for Built Environment Indicators and Neighborhood Racial Topology

	% Dilapidated			*% Green Space*			*% Crosswalk*			*% Non-Single Family Home*			*% Single Lane*		
Predictors	*Coef*	*CI*	*p*	*Coef*	*CI*	*p*	*Coef*	*CI*	*p*	*Coef*	*CI*	*p*	*Coef*	*CI*	*p*
Intercept	0.22	0.21 – 0.24	<0.01	0.89	0.87 – 0.91	<0.01	0.03	0.02 – 0.03	<0.01	0.22	0.19 – 0.25	<0.01	0.70	0.69 – 0.72	<0.01
Racial Composition (Ref: Predominantly White)															
Unclassified	0.03	0.03 – 0.04	<0.01	-0.07	-0.07 – -0.07	<0.01	0.02	0.02 – 0.02	<0.01	0.13	0.13 – 0.13	<0.01	-0.04	-0.04 – -0.03	<0.01
Predominantly Black	0.02	0.02 – 0.02	<0.01	-0.09	-0.09 – -0.08	<0.01	0.02	0.02 – 0.02	<0.01	0.18	0.18 – 0.19	<0.01	0.00	-0.00 – 0.01	0.47
Predominantly minoritized racial group other than Black	0.07	0.07 – 0.08	<0.01	-0.18	-0.18 – -0.17	<0.01	0.03	0.03 – 0.03	<0.01	0.25	0.25 – 0.26	<0.01	-0.08	-0.08 – -0.07	<0.01
Random Effects															
σ^2	0.01			0.02			0.00			0.04			0.02		
τ_{00}	0.00 $_{State}$			0.01 $_{State}$			0.00 $_{State}$			0.01 $_{State}$			0.00 $_{State}$		
ICC	0.29			0.25			0.23			0.20			0.13		
N	51 $_{State}$			51 $_{State}$			51 $_{State}$			51 $_{State}$			51 $_{State}$		
Observations	56812			56818			56818			56818			56818		
Marginal R^2 / Conditional R^2	0.035 / 0.314			0.111 / 0.331			0.053 / 0.271			0.121 / 0.298			0.028 / 0.157		

© 2023 Yang Y et al. *JAMA Network Open*.

eAppendix 6. Mediation Analysis

eFigure 5. Estimates and 95% CIs of Average Causal Mediation Effects (ACME) and Percent Mediated of the Association Between Neighborhood Racial Topology and Health Outcomes Mediated by Built Environment Indicators

© 2023 Yang Y et al. *JAMA Network Open*.

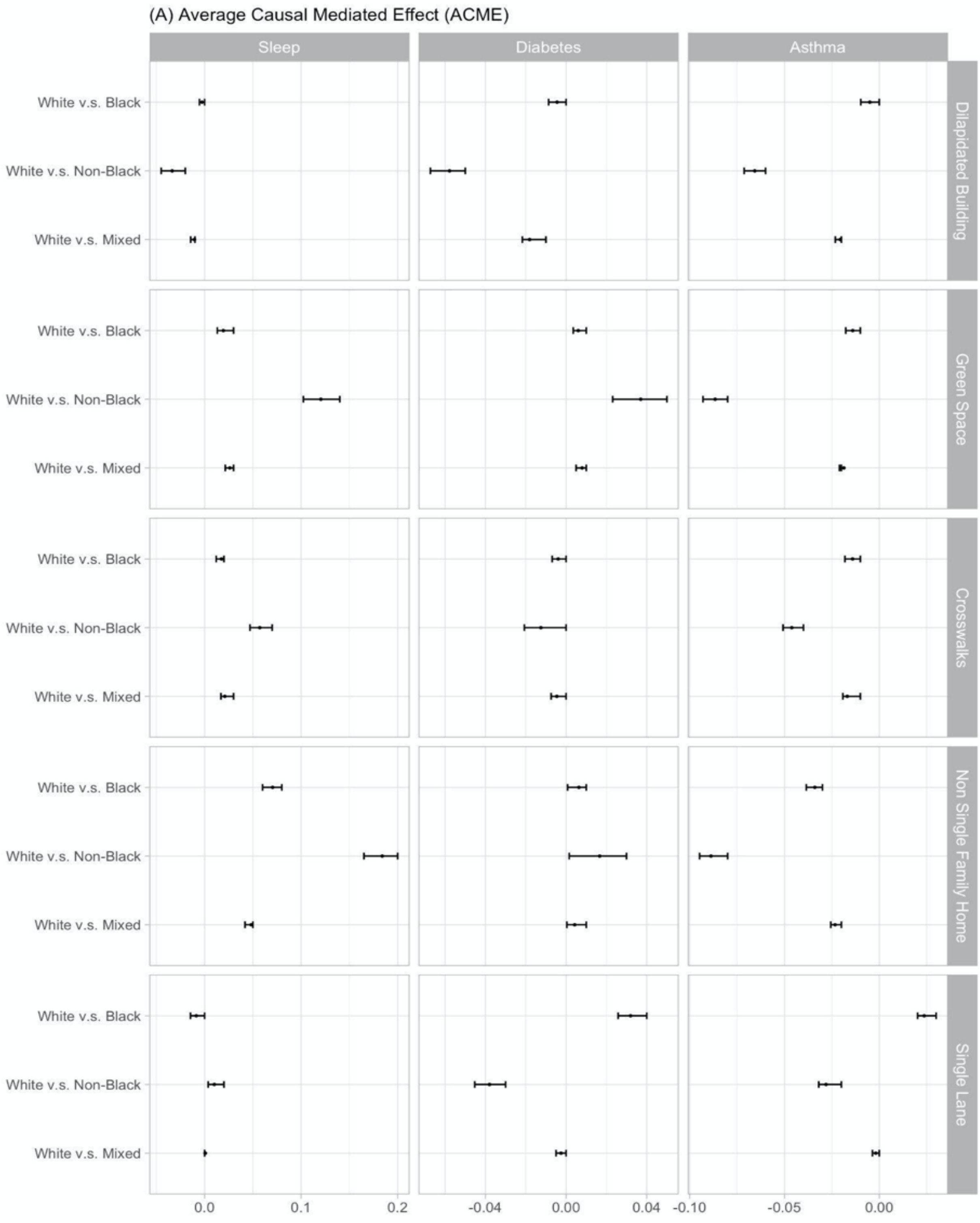

© 2023 Yang Y et al. *JAMA Network Open*.

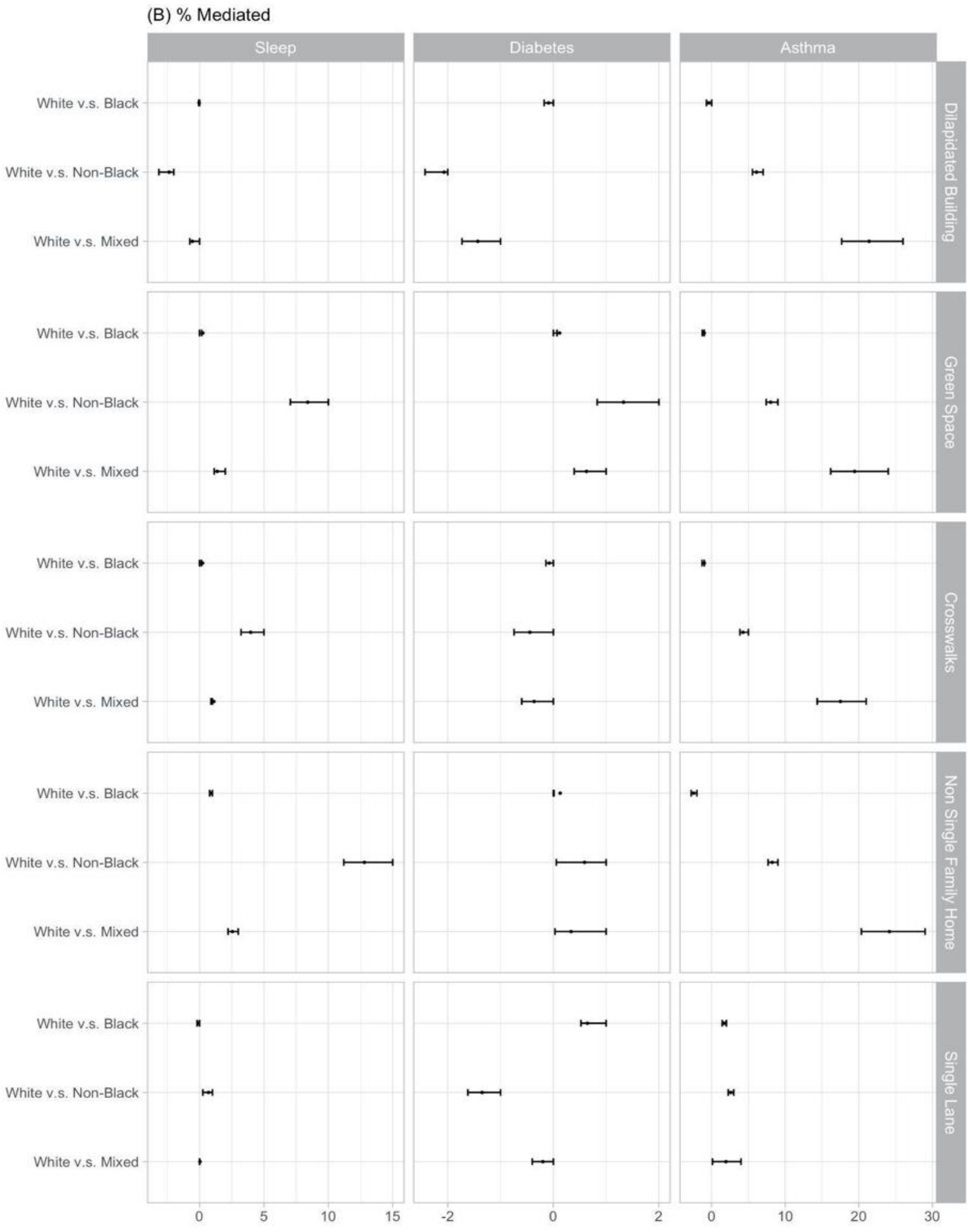

© 2023 Yang Y et al. *JAMA Network Open*.

eAppendix 7. HOLC Analysis

We conducted preliminary analysis to measure the similarity between HOLC grades and our neighborhood racial topology.

eTable 3 shows that for all the A grade tracts, we classified 64% as predominantly White neighborhoods and only 8% as neighborhoods with predominantly Black or predominantly minoritized racial group other than Black. For B grade tracts, the majority were either predominantly White or unclassified neighborhoods. Furthermore, for tracts that were graded as D, 26% were predominantly Black neighborhoods and 44% were unclassified. From grades A to D, the percent of tracts classified as predominantly White neighborhoods declined, and the percent classified as predominantly Black and predominantly minoritized racial groups other than Black neighborhoods increased.

eTable 3. Percent Distribution of Neighborhood Racial Topology by HOLC Grades

	A	B	C	D	Other
Predominantly White			0.22	0.14	
Unclassified	0.26				
Predominantly Black	0.08	0.15	0.17	0.26	0.10
Predominantly minoritized racial group other than Black	0.01	0.07	0.16	0.16	0.08
Total	1	1	1	1	1

A Chi-Squared test to measure association between our neighborhood racial topology and the HOLC grades was statistically significant (*X-squared* = 1299.2, $p < 0.001$).

© 2023 Yang Y et al. *JAMA Network Open*.

eTable 4. Hierarchical Linear Model (HLM) Regression With State Random Intercepts for HOLC Grades

	% Dilapidated			*% Green Space*			*% Crosswalk*			*% Non-Single Family Home*			*% Single Lane*		
Predictors	Coef	*CI*	*p*	Coef	*CI*	*p*	Coef	*CI*	*p*	Coef	*CI*	*p*	Coef	*CI*	*p*
Intercept	0.21	0.19 – 0.24	<0.001	0.96	0.93 – 0.99	<0.001	0.04	0.03 – 0.05	<0.001	0.21	0.17 – 0.26	<0.001	0.77	0.75 – 0.80	<0.001
HOLC Grades (Ref: A (green)—"best")															
B (blue)—"still desirable"	0.03	0.02 – 0.04	<0.001	-0.07	-0.09 – -0.06	<0.001	0.01	0.01 – 0.02	<0.001	0.12	0.09 – 0.15	<0.001	-0.03	-0.04 – -0.01	0.002
C (yellow)—"definitely declining"	0.04	0.03 – 0.05	<0.001	-0.15	-0.16 – -0.13	<0.001	0.02	0.01 – 0.02	<0.001	0.22	0.19 – 0.24	<0.001	-0.07	-0.08 – -0.05	<0.001
D (red)—"hazardous"	0.05	0.04 – 0.06	<0.001	-0.22	-0.23 – -0.20	<0.001	0.03	0.02 – 0.03	<0.001	0.34	0.31 – 0.36	<0.001	-0.15	-0.17 – -0.14	<0.001
Other	0.03	0.02 – 0.04	<0.001	-0.11	-0.12 – -0.09	<0.001	0.00	-0.00 – 0.01	0.435	0.10	0.08 – 0.13	<0.001	-0.08	-0.10 – -0.07	<0.001
Random Effects															
σ^2	0.01			0.02			0.00			0.06			0.02		
τ_{00}	0.01 State			0.01 State			0.00 State			0.01 State			0.00 State		
ICC	0.37			0.19			0.20			0.20			0.17		
N	39 State			39 State			39 State			39 State			39 State		
Observations	14264			14265			14265			14265			14265		
Marginal R^2 / Conditional R^2	0.006 / 0.369			0.078 / 0.258			0.030 / 0.226			0.106 / 0.281			0.050 / 0.215		

© 2023 Yang Y et al. *JAMA Network Open*.

eTable 5. Hierarchical Linear Model (HLM) Regression With State Random Intercepts for HOLC Grades With Socioeconomic Covariates

	% Dilapidated			*% Green Space*			*% Crosswalk*			*% Non-Single Family Home*			*% Single Lane*		
Predictors	Coef	*CI*	*p*	Coef	*CI*	*p*	Coef	*CI*	*p*	Coef	*CI*	*p*	Coef	*CI*	*p*
Intercept	0.35	0.27 – 0.43	<0.001	0.86	0.74 – 0.97	<0.001	0.07	0.03 – 0.11	<0.001	0.62	0.46 – 0.78	<0.001	0.76	0.65 – 0.86	<0.001
HOLC Grades (Ref: A (green)—"best")															
B (blue)—"still desirable"	0.02	0.00 – 0.03	0.004	-0.02	-0.04 – -0.01	0.004	0.01	0.01 – 0.01	<0.001	0.07	0.05 – 0.09	<0.001	-0.00	-0.01 – 0.01	0.890
C (yellow)—"definitely declining"	0.02	0.01 – 0.03	<0.001	-0.05	-0.07 – -0.04	<0.001	0.01	0.01 – 0.02	<0.001	0.11	0.09 – 0.13	<0.001	-0.02	-0.03 – -0.01	0.002
D (red)—"hazardous"	0.03	0.02 – 0.04	<0.001	-0.11	-0.12 – -0.09	<0.001	0.02	0.01 – 0.02	<0.001	0.18	0.16 – 0.20	<0.001	-0.08	-0.10 – -0.07	<0.001
Other	0.02	0.01 – 0.03	<0.001	-0.05	-0.07 – -0.04	<0.001	0.00	-0.00 – 0.01	0.503	0.06	0.04 – 0.08	<0.001	-0.06	-0.07 – -0.05	<0.001
log(Household Income)	-0.00	-0.01 – 0.01	0.826	-0.05	-0.06 – -0.04	<0.001	0.00	-0.00 – 0.01	0.062	0.04	0.03 – 0.05	<0.001	-0.03	-0.04 – -0.03	<0.001
% College	0.00	-0.01 – 0.01	0.831	0.09	0.07 – 0.11	<0.001	0.04	0.04 – 0.05	<0.001	0.20	0.17 – 0.22	<0.001	-0.08	-0.10 – -0.06	<0.001
Age	0.00	-0.00 – 0.00	0.109	-0.00	-0.00 – 0.00	0.117	0.00	0.00 – 0.00	<0.001	0.00	0.00 – 0.00	<0.001	-0.00	-0.00 – -0.00	<0.001
% Female	-0.03	-0.07 – 0.00	0.060	0.32	0.27 – 0.37	<0.001	-0.03	-0.05 – -0.02	<0.001	-0.35	-0.42 – -0.28	<0.001	0.23	0.19 – 0.28	<0.001
% Insured	-0.03	-0.06 – 0.01	0.116	0.25	0.20 – 0.29	<0.001	-0.02	-0.03 – -0.00	0.037	-0.39	-0.46 – -0.33	<0.001	0.11	0.07 – 0.15	<0.001
% Owner	-0.11	-0.12 – -0.10	<0.001	0.32	0.31 – 0.34	<0.001	-0.10	-0.11 – -0.09	<0.001	-0.74	-0.76 – -0.71	<0.001	0.45	0.44 – 0.47	<0.001
% Vacant	-0.11	-0.13 – -0.09	<0.001	-0.15	-0.18 – -0.12	<0.001	-0.01	-0.02 – -0.00	0.019	0.21	0.17 – 0.25	<0.001	0.04	0.02 – 0.07	0.001
% Single Family	-0.04	-0.05 – -0.03	<0.001	0.05	0.03 – 0.06	<0.001	-0.01	-0.01 – -0.00	<0.001	-0.06	-0.08 – -0.04	<0.001	0.06	0.05 – 0.07	<0.001
Random Effects															
σ^2	0.01			0.02			0.00			0.03			0.01		
τ_{00}	0.00 State			0.00 State			0.00 State			0.01 State			0.00 State		
ICC	0.34			0.22			0.19			0.27			0.17		
N	39 State			39 State			39 State			39 State			39 State		
Observations	14264			14265			14265			14265			14265		
Marginal R^2 / Conditional R^2	0.051 / 0.376			0.262 / 0.426			0.198 / 0.352			0.436 / 0.587			0.360 / 0.470		

© 2023 Yang Y et al. *JAMA Network Open*.

eAppendix 8. Neighborhood and Sensitivity Analysis
We obtained data for six single-race racial and ethnic categories, namely Non-Hispanic White, Non-Hispanic Black, Asian, American Indian and Alaska Native, Native Hawaiian and Other Pacific Islander, and Hispanics (of any race) from the 2019 American Community Survey (ACS) 5-year estimates.[12]

We assessed neighborhood disparities in built environment indicators using two approaches: racial majority tracts and HOLC grades. There is no consensus on how to define the racial majority in a neighborhood. Recent studies defined the racial majority as the racial/ethnic group that represents at least fifty percent of the neighborhood population.[13–15] We therefore used this criteria of at least fifty percent majority for all racial/ethnic groups except white populations and performed a sensitivity analysis to assess the robustness of our findings to varied definitions.

Specifically, we categorized the racial majority in a neighborhood using the same approach as Gibbons[15]:

- Predominantly White: Census tracts that had at least 60% of non-Hispanic White residents and none of the racial minority groups made up 20% of the population.
- Predominantly Black: Census tracts that had at least 50% of the population identified as non-Hispanic Black residents and the second largest racial group made up less than 20% of the population.
- Predominantly minoritized racial group other than Black: Census tracts that had at least 50% of the population identified as any of the racial minority groups other than non-Hispanic Black, and the second largest racial group made up less than 20% of the population.
- Unclassified: Census tracts that could not be classified as any of the topologies above.

Gibbons[15] used 20% cut-off because 20% is roughly the upper bound for the average population makeup for minoritized racial groups in the U.S.[15,16]

For the analysis using HOLC grades, we used the redlining census tract crosswalk data from Mapping Inequality.[17] There are challenges to matching present day census tracts to HOLC grades. One study reported that about 60% of 2010 census tracts cross HOLC areas with multiple grades.[18] Given that there is no consensus on how best to assign HOLC grades to census tracts, we chose to adopt the approach used by Krieger et al.[19] For each census tract, we determined the percent of the geographical area assigned to each grade. Then we assigned the tract the grade representing at least 50% of the area. If no HOLC grade was assigned to at least 50% of the census tract, the census tract was labeled "other". This resulted in 2,381 grade D, 3,847 grade C, 1,562 grade B, 425 grade A and 6,796 other. The remaining 44,228 census tracts in our data could not be assigned a grade.

Sensitivity Analysis

© 2023 Yang Y et al. *JAMA Network Open*.

To test the robustness of our neighborhood racial topology definitions, we further experimented with different thresholds. We defined three ways to categorize census tracts.

1. No cut-off
    a. Predominantly White: Tracts that had at least 60% of non-Hispanic White residents.
    b. Predominantly Black: Tracts that had at least 50% non-Hispanic Black residents
    c. Predominantly minoritized racial group other than Black: Tracts that consisted of at least 50% of a minority racial/ethnic group other than Black.
    d. Unclassified: Tracts that could not be classified into any of the typologies above.
2. 20% cut off (our approach):
    a. Predominantly White: Tracts that had at least 60% of non-Hispanic White residents and no minority group represented more than 20%.
    b. Predominantly Black: Tracts that had at least 50% non-Hispanic Black residents and no more than 20% of another racial/ethnic group.
    c. Predominantly minoritized racial group other than Black: Tracts that consisted of at least 50% of a certain minority racial/ethnic group other than Black and no more than 20% of non-Hispanic Black residents.
    d. Unclassified: Tracts that could not be classified into any of the typologies above.
3. 10% cut off:
    a. Predominantly White: Tracts that had at least 60% of non-Hispanic White residents and no minority group represented more than 10%.
    b. Predominantly Black: Tracts that had at least 50% non-Hispanic Black residents and no more than 10% of another racial/ethnic group.
    c. Predominantly minoritized racial group other than Black: Tracts that consisted of at least 50% of a certain minority race/ethnic group other than Black and no more than 10% of non-Hispanic Black residents.
    d. Unclassified: Tracts that could not be classified into any of the typologies above.

The number of tracts falling into different categories changed based on the definition; with larger thresholds, fewer tracts were categorized as predominantly White neighborhoods, and more tracts were defined as unclassified neighborhoods. The numbers for each category are shown in Table S6.

We then ran the HLM models with the same specification for different neighborhood racial topologies (see Tables S7 and S8). In general, coefficients were consistent across the different neighborhood racial topologies. Except for *% Single Lane*, all of the coefficients were in the same direction. No cut-off and 20% cut-off models had negative coefficients for neighborhoods with Unclassified racial composition (-0.01 and -0.00, respectively) whereas the 10% cut-off model had a positive coefficient (0.01).

eTable 6. Number of Tracts by Different Racial Topologies

Approach	Predominantly White (N)	Unclassified (N)	Predominantly Black (N)	Predominantly minoritized racial group other than Black (N)
No cut off	32,748	13,774	5,486	7,223
20% cut off	28,940	22,465	3,467	4,539
10% cut off	18,048	37,145	2,015	2,023

The values of ICCs were similar across the neighborhood racial topologies although the no cut-off model had slightly smaller ICCs. For *% Dilapidated,* approximately 28% of the variance could be

© 2023 Yang Y et al. *JAMA Network Open*.

explained by state differences under the no cut-off model, while 29% of the variance was at the state level under the 20% and 10% cut-off models. Similarly, 26% of the variance in the green space model was at the state level for the no cut-off model, whereas 27% of the variance was at the state level for both 20% and 10% cut-off models. Similar patterns were noted in *% Non-single family home*: the ICC for the no-cut off model was 0.24 while the ICCs for the 20% and 10% cut-off models were both 0.25.

For the effect sizes, neighborhoods with predominantly minoritized racial group other than Black under the 10% cut-off model, overall, had slightly larger coefficients than those of no cut-off and 20% cut-off models. For example, for *% Dilapidated*, coefficients for this neighborhoods were 0.05, 0.06, and 0.07, respectively, for no cut-off, 20% cut-off and 10% cut-off models. Similarly, for *% Green Space*, coefficients ranged from -0.10 to -0.12. Neighborhoods with unclassified racial composition had stable coefficients across the three models for all of the built environment indicators. For predominantly Black neighborhoods, the effect size was generally similar between the 20% and 10% cut-off models.

Furthermore, the model fit was consistent across the three definitions. The marginal R-squared demonstrates the proportion of variance explained by the fixed effect whereas the conditional R-squared demonstrates the proportion of variance explained by both the fixed and random effects. Regarding the full models, the 20% cut-off models, in general, had the highest conditional R-squared across all built environment indicators. No cut-off models, on the other hand, had the highest marginal R-squared. For instance, for *% Dilapidated*, the values of conditional R-squared were as follows: 0.350, 0.354, and 0.353, respectively for no cut-off, 20% cut-off and 10% cut-off models. The values of marginal R-squared for the same built environment indicator were as follows: 0.092, 0.091, and 0.085. Overall, our sensitivity analysis suggested our models were robust to the three neighborhood racial topology definitions.

© 2023 Yang Y et al. *JAMA Network Open*.

eTable 7. Hierarchical Linear Model (HLM) Regression With State Random Intercepts Using the No Cutoff For Racial Composition

	% Dilapidated			*% Green Space*			*% Crosswalk*			*% Non-Single Family Home*			*% Single Lane*		
Predictors	*Coef*	*CI*	*p*	*Coef*	*CI*	*p*	*Coef*	*CI*	*p*	*Coef*	*CI*	*p*	*Coef*	*CI*	*p*
Intercept	0.20	0.15 – 0.25	<0.001	0.86	0.80 – 0.91	<0.001	0.12	0.11 – 0.14	<0.001	0.69	0.61 – 0.77	<0.001	0.80	0.74 – 0.86	<0.001
Racial Composition (Ref: Predominantly White)															
Unclassified	0.02	0.02 – 0.02	<0.001	-0.02	-0.03 – -0.02	<0.001	0.01	0.01 – 0.01	<0.001	0.04	0.03 – 0.04	<0.001	-0.01	-0.01 – -0.00	<0.001
Predominantly Black	0.00	-0.00 – 0.01	0.23	-0.01	-0.02 – -0.01	<0.001	0.00	0.00 – 0.01	<0.001	0.05	0.05 – 0.06	<0.001	0.03	0.02 – 0.03	<0.001
Predominantly minoritized racial group other than Black	0.05	0.05 – 0.05	<0.001	-0.10	-0.10 – -0.09	<0.001	0.02	0.02 – 0.02	<0.001	0.14	0.14 – 0.15	<0.001	-0.04	-0.04 – -0.03	<0.001
Covariates															
log(Household Income)	0.00	-0.00 – 0.01	0.10	-0.01	-0.02 – -0.01	<0.001	-0.01	-0.01 – -0.00	<0.001	-0.01	-0.01 – -0.00	0.05	-0.03	-0.03 – -0.02	<0.001
% College	0.05	0.04 – 0.06	<0.001	0.07	0.06 – 0.08	<0.001	0.05	0.05 – 0.05	<0.001	0.16	0.15 – 0.17	<0.001	0.01	-0.00 – 0.02	0.13
Age	0.00	-0.00 – 0.00	0.52	0.00	0.00 – 0.00	<0.001	0.00	0.00 – 0.00	<0.001	0.00	0.00 – 0.00	<0.001	-0.00	-0.00 – -0.00	<0.001
% Female	0.07	0.05 – 0.10	<0.001	0.17	0.14 – 0.19	<0.001	-0.01	-0.02 – -0.00	0.02	-0.28	-0.32 – -0.25	<0.001	0.23	0.20 – 0.25	<0.001
% Insured	0.04	0.02 – 0.05	<0.001	-0.09	-0.11 – -0.07	<0.001	0.00	-0.00 – 0.01	0.32	0.02	-0.01 – 0.05	0.12	-0.08	-0.11 – -0.06	<0.001
% Owner	-0.13	-0.13 – -0.12	<0.001	0.22	0.21 – 0.22	<0.001	-0.09	-0.09 – -0.09	<0.001	-0.57	-0.58 – -0.56	<0.001	0.33	0.33 – 0.34	<0.001
% Vacant	-0.07	-0.08 – -0.06	<0.001	-0.16	-0.18 – -0.15	<0.001	-0.01	-0.01 – -0.00	<0.001	0.29	0.28 – 0.31	<0.001	-0.12	-0.13 – -0.10	<0.001
% Single Family	-0.01	-0.02 – -0.00	<0.001	0.02	0.01 – 0.02	<0.001	-0.01	-0.01 – -0.00	<0.001	-0.06	-0.07 – -0.05	<0.001	0.08	0.07 – 0.08	<0.001
Random Effects															
σ^2	0.01			0.01			0.00			0.03			0.01		
τ_{00}	0.00 State			0.00 State			0.00 State			0.01 State			0.00 State		
ICC	0.28			0.26			0.23			0.24			0.13		
N	51 State			51 State			51 State			51 State			51 State		
Observations	56812			56818			56818			56818			56818		
Marginal R^2 / Conditional R^2	0.092 / 0.350			0.205 / 0.411			0.262 / 0.430			0.384 / 0.532			0.205 / 0.306		

© 2023 Yang Y et al. *JAMA Network Open*.

eTable 8. Hierarchical Linear Model (HLM) Regression With State Random Intercepts Using a 10% Cutoff for Racial Composition

	% Dilapidated			*% Green Space*			*% Crosswalk*			*% Non-Single Family Home*			*% Single Lane*		
Predictors	*Coef*	*CI*	*p*	*Coef*	*CI*	*p*	*Coef*	*CI*	*p*	*Coef*	*CI*	*p*	*Coef*	*CI*	*p*
Intercept	0.22	0.17 – 0.26	<0.001	0.79	0.74 – 0.85	<0.001	0.13	0.12 – 0.15	<0.001	0.79	0.71 – 0.87	<0.001	0.76	0.71 – 0.82	<0.001
Racial Composition (Ref: Predominantly White)															
Unclassified	0.02	0.01 – 0.02	<0.001	-0.02	-0.02 – -0.01	<0.001	0.01	0.01 – 0.01	<0.001	0.03	0.03 – 0.03	<0.001	0.01	0.01 – 0.02	<0.001
Predominantly Black	-0.00	-0.01 – 0.00	0.29	-0.01	-0.02 – -0.01	<0.001	0.01	0.00 – 0.01	<0.001	0.06	0.05 – 0.06	<0.001	0.06	0.05 – 0.07	<0.001
Predominantly minoritized racial group other than Black	0.07	0.06 – 0.07	<0.001	-0.12	-0.12 – -0.11	<0.001	0.02	0.01 – 0.02	<0.001	0.17	0.16 – 0.18	<0.001	-0.03	-0.04 – -0.03	<0.001
Covariates															
log(Household Income)	0.01	0.00 – 0.01	0.01	-0.02	-0.02 – -0.01	<0.001	-0.00	-0.01 – -0.00	<0.001	-0.00	-0.01 – 0.00	0.49	-0.03	-0.03 – -0.02	<0.001
% College	0.04	0.03 – 0.05	<0.001	0.08	0.07 – 0.09	<0.001	0.05	0.04 – 0.05	<0.001	0.14	0.12 – 0.15	<0.001	0.01	0.01 – 0.02	<0.001
Age	-0.00	-0.00 – 0.00	0.25	0.00	0.00 – 0.00	<0.001	0.00	0.00 – 0.00	<0.001	0.00	0.00 – 0.00	<0.001	-0.00	-0.00 – -0.00	<0.001
% Female	0.07	0.05 – 0.09	<0.001	0.18	0.15 – 0.20	<0.001	-0.01	-0.02 – -0.00	0.02	-0.28	-0.32 – -0.25	<0.001	0.23	0.21 – 0.26	<0.001
% Insured	0.00	-0.02 – 0.02	0.81	-0.01	-0.03 – 0.01	0.26	-0.01	-0.02 – -0.01	<0.001	-0.10	-0.13 – -0.07	<0.001	-0.04	-0.06 – -0.02	<0.001
% Owner	-0.13	-0.14 – -0.12	<0.001	0.22	0.21 – 0.23	<0.001	-0.09	-0.09 – -0.09	<0.001	-0.58	-0.59 – -0.57	<0.001	0.34	0.33 – 0.35	<0.001
% Vacant	-0.07	-0.08 – -0.06	<0.001	-0.16	-0.17 – -0.14	<0.001	-0.01	-0.01 – -0.01	<0.001	0.28	0.27 – 0.30	<0.001	-0.11	-0.13 – -0.10	<0.001
% Single Family	-0.01	-0.02 – -0.00	<0.001	0.02	0.01 – 0.03	<0.001	-0.01	-0.01 – -0.00	<0.001	-0.05	-0.06 – -0.04	<0.001	0.08	0.07 – 0.08	<0.001
Random Effects															
σ^2	0.01			0.01			0.00			0.03			0.01		
τ_{00}	0.00 State			0.01 State			0.00 State			0.01 State			0.00 State		
ICC	0.29			0.27			0.23			0.25			0.13		
N	51 State			51 State			51 State			51 State			51 State		
Observations	56812			56818			56818			56818			56818		
Marginal R^2 / Conditional R^2	0.085 / 0.353			0.187 / 0.407			0.253 / 0.426			0.366 / 0.526			0.196 / 0.304		

© 2023 Yang Y et al. *JAMA Network Open*.

eTable 9. Model Performance Comparison (Marginal R^e/Conditional R^2)

	% Dilapidated		*% Green Space*		*% Crosswalk*		*% Non-Single Family Home*		*% Single Lane*	
	Race Only	Full Model	Race Only	Full Model	Race Only	Full Model	Race Only	Full Model	Race Only	Full Model
No cut-off	0.037 / 0.309	0.092 / 0.350	0.116 / 0.329	0.205 / 0.411	0.056 / 0.270	0.262 / 0.430	0.126 / 0.297	0.384 / 0.532	0.033 / 0.157	0.205 / 0.306
20% cut off	0.035 / 0.314	0.091 / 0.354	0.111 / 0.331	0.202 / 0.415	0.053 / 0.271	0.260 / 0.430	0.121 / 0.298	0.380 / 0.533	0.028 / 0.157	0.202 / 0.306
10% cut off	0.032 / 0.314	0.085 / 0.353	0.076 / 0.305	0.187 / 0.407	0.051 / 0.267	0.253 / 0.426	0.097 / 0.275	0.366 / 0.526	0.016 / 0.148	0.196 / 0.304

© 2023 Yang Y et al. *JAMA Network Open*.

eReferences.

1. Kaufman L, Rousseeuw PJ. Clustering Large Applications (Program CLARA). In: *Finding Groups in Data*. John Wiley & Sons, Ltd; 1990:126-163. doi:10.1002/9780470316801.ch3

2. Kaufman L, Rousseeuw PJ. *Finding Groups in Data: An Introduction to Cluster Analysis*. John Wiley & Sons; 2009.

3. Tibshirani R, Walther G, Hastie T. Estimating the number of clusters in a data set via the gap statistic. *Journal of the Royal Statistical Society: Series B (Statistical Methodology)*. 2001;63(2):411-423.

4. Nguyen TT, Nguyen QC, Rubinsky AD, et al. Google Street View-Derived Neighborhood Characteristics in California Associated with Coronary Heart Disease, Hypertension, Diabetes. *IJERPH*. 2021;18(19):10428. doi:10.3390/ijerph181910428

5. Nguyen QC, Belnap T, Dwivedi P, et al. Google Street View Images as Predictors of Patient Health Outcomes, 2017–2019. *BDCC*. 2022;6(1):15. doi:10.3390/bdcc6010015

6. Yue X, Antonietti A, Alirezaei M, et al. Using Convolutional Neural Networks to Derive Neighborhood Built Environments from Google Street View Images and Examine Their Associations with Health Outcomes. *International Journal of Environmental Research and Public Health*. 2022;19(19):12095.

7. Olivoto T. metan: Multi Environment Trials Analysis. Published online June 10, 2022. Accessed November 15, 2022. https://CRAN.R-project.org/package=metan

8. Bates D, Maechler M, Bolker [aut B, et al. lme4: Linear Mixed-Effects Models using “Eigen” and S4. Published online July 8, 2022. Accessed September 6, 2022. https://CRAN.R-project.org/package=lme4

9. Maechler M, original) PR (Fortran, original) AS (S, et al. cluster: “Finding Groups in Data”: Cluster Analysis Extended Rousseeuw et al. Published online August 22, 2022. Accessed September 6, 2022. https://CRAN.R-project.org/package=cluster

10. Tingley D, Yamamoto T, Hirose K, Keele L, Imai K. Mediation: R package for causal mediation analysis. Published online 2014.

11. Clarke P. When can group level clustering be ignored? Multilevel models versus single-level models with sparse data. *Journal of Epidemiology & Community Health*. 2008;62(8):752-758.

12. US Census Bureau. American Community Survey 5-Year Data (2009-2020). Census.gov. Accessed July 1, 2022. https://www.census.gov/data/developers/data-sets/acs-5year.html

13. Do DP, Frank R. Unequal burdens: assessing the determinants of elevated COVID-19 case and death rates in New York City’s racial/ethnic minority neighbourhoods. *J Epidemiol Community Health*. 2021;75(4):321-326.

© 2023 Yang Y et al. *JAMA Network Open*.

14. Liu D, Kwan MP, Kan Z, Song Y, Li X. Racial/Ethnic Inequity in Transit-Based Spatial Accessibility to COVID-19 Vaccination Sites. *Journal of Racial and Ethnic Health Disparities*. Published online 2022:1-9.

15. Gibbons J. Neighborhood Racial/Ethnic Composition and Medical Discrimination's Relation to Mammograms: A Philadelphia Case Study. *Race Soc Probl*. 2021;13(3):234-244. doi:10.1007/s12552-021-09312-9

16. Gibbons J, Yang TC. Connecting across the divides of race/ethnicity: How does segregation matter? *Urban Affairs Review*. 2016;52(4):531-558.

17. "Mapping Inequality," American Panorama, ed. Robert K. Nelson and Edward L. Ayers. Published online November 14, 2022. https://dsl.richmond.edu/panorama/redlining/#loc=13/42.323/-71.185&city=brookline-ma&text=downloads

18. Noelke C, Outrich M, Baek M, et al. Connecting past to present: Examining different approaches to linking historical redlining to present day health inequities. *Plos one*. 2022;17(5):e0267606.

19. Krieger N, Van Wye G, Huynh M, et al. Structural racism, historical redlining, and risk of preterm birth in New York City, 2013–2017. *American journal of public health*. 2020;110(7):1046-1053.

© 2023 Yang Y et al. *JAMA Network Open*.
