# Supplementary material for: Association of Neighborhood Racial and Ethnic Composition and Historical Redlining With Built Environment Indicators Derived From Street View Images in the US
Source: JAMA Netw Open. 2023 Jan 18;6(1):e2251201. doi: 10.1001/jamanetworkopen.2022.51201 (PMC9856713; doi:10.1001/jamanetworkopen.2022.51201)
Supplement: Supplement 2. — Data Sharing Statement [file jamanetwopen-e2251201-s002.pdf]

Data Sharing Statement

Yang. Association of Neighborhood Racial and Ethnic Composition and Historical Redlining With Built Environment Indicators Derived From Street-View Images in the US. *JAMA Netw Open*. Published January 18, 2023. doi:10.1001/jamanetworkopen.2022.51201

Data
Data available: No

Additional Information
Explanation for why data not available: The links to data sources, data files and code used in the study can be accessed on Github: https://github.com/BU-Center-for-Antiracist-Research/neighborhood_racial_composition_built_environment_and_health_outcome.
